# Influenza neuraminidase active site proximity assay for rapid profiling of inhibitory antibodies and antigenic drift

**DOI:** 10.1038/s41541-025-01173-2

**Published:** 2025-06-07

**Authors:** Jin Gao, Galina Landgraf, Yue Yuan, Hai Yu, Soma Saeidi, Hyeog Kang, Mira Rakic Martinez, Luca Giurgea, Vladimir Lugovtsev, Jason Gorman, Matthew Memoli, Xi Chen, Zhiping Ye, Robert Daniels

**Affiliations:** 1 Division of Viral Products, Center for Biologics Evaluation and Research, Food and Drug Administration, Silver Spring, MD USA; 2 Department of Chemistry, University of California, One Shields Avenue, Davis, CA USA; 3 Laboratory of Infectious Diseases Clinical Studies Unit, Laboratory of Infectious Diseases, National Institute of Allergy and Infectious Diseases, National Institutes of Health, Bethesda, MD USA

**Keywords:** Influenza virus, Viral host response, Influenza virus

## Abstract

Efficient approaches that can help to select vaccine strains for the influenza virus neuraminidase (NA) antigen are currently needed to advance the development of vaccines containing NA. Here, we present a rapid and cost-effective solution-based NA active site proximity assay (NASPA) for measuring NA activity inhibitory (NAI) antibodies. This simplified assay uses large “bulky” NA active site-binding inhibitors to replace the sialylated glycoprotein substrates in common NA enzyme-linked lectin assay (ELLA) approaches. Our results with ferret antisera and monoclonal antibodies against vaccine strain NAs show a strong correlation between NASPA and ELLA titers, and that NASPA titers are not influenced by anti-HA antibodies. Consequently, NASPA can be used with influenza A or B strains and with the latter it revealed incremental antigenic changes in the NAs from recent B Victoria lineage vaccine strains. By coupling NASPA with a simple activity assay, we also found that steric and active site-binding NAI antibodies against circulating NAs are common in adult human sera. Finally, we demonstrate that NASPA can be modified by incorporating novel NA substrate-analog-based inhibitors. Together, these results suggest that NASPA can aid the development of vaccines containing NA by helping to select suitable vaccine strains and profile anti-NA antibody responses.

## Introduction

Influenza virus infections are largely orchestrated by the viral envelope glycoproteins hemagglutinin (HA) and neuraminidase (NA). The more abundant HA glycoprotein facilitates cell entry by binding to receptors on the cell surface that contain a terminal sialic acid residue and fusing the viral and endosomal membranes following endocytosis^1^. In contrast, NA is a Ca^2+^-dependent enzyme that promotes viral movement by removing local sialic acid residues that can result in persistent HA binding^2,3^. NA catalyzes the removal by hydrolyzing the sialyl glycosidic linkage with the underlying sugar molecules, which are often galactosides^4,5^. While the NA active site has been one of the primary targets for influenza antiviral (e.g., oseltamivir, zanamivir, and peramivir) development^6^, influenza vaccines have historically focused on eliciting antibodies that inhibit the receptor binding function of HA^7^.

Currently, many different vaccine strategies are being pursued to improve the breadth and efficacy of seasonal influenza vaccines^8–12^. One of these involves developing approaches for eliciting protective antibody responses against NA in addition to HA^13–20^. Supporting this strategy, numerous studies have shown that NA antigens from a variety of sources can elicit anti-NA antibodies that are capable of reducing the severity of influenza infections in both humans^21,22^ and animal models^13,16,18,23–26^. Furthermore, several clinical studies have indicated that NA activity inhibitory (NAI) antibody responses may correlate with protection^27–29^, although it is not clear if this correlation applies to NAI antibodies that function *via* steric interference, active site-binding, or both. In addition, HA and NA antigenic drift has been shown to be discordant^30^, implying anti-NA antibody responses may help to increase the breadth of protection provided by influenza vaccines^10,31,32^. However, a strain selection framework is currently only established for HA antigens, making it difficult to identify appropriate NAs for these new vaccine strategies.

Vaccine strains for HA are selected based on a combination of genetic and antigenic data from circulating strains along with their prevalence and location^33^. During this selection process, NA genetic data is also often obtained, but little to no antigenic data is collected. HA antigenicity is commonly assessed using ferret antisera in hemagglutination inhibition (HI) assays or high-content imaging-based neutralization tests (HINT). A similar approach has been taken for NA that uses ferret antisera to monitor changes in NAI antibody titers^34–36^. However, these NAI titers have generally been obtained from a fetuin-based enzyme-linked lectin assay (ELLA) that is susceptible to interference (false positives) from antibodies that recognize the HA in the test virus^37–39^. To minimize this issue, several ELLA modifications have been implemented. These include using reassortant test viruses that contain an HA subtype such as H6 or H7 that should not be recognized by antibodies in the serum^40,41^, detergent-treated viruses^42^, and recombinant NA^43^. However, these approaches further increase the time, labor, and complexity, making it expensive and difficult to use ELLA for the antigenic analysis of NA in many strains each season.

The lack of an approach that can easily be implemented to identify suitable vaccine strains for NA antigens remains a major barrier for generating vaccines that can elicit protective responses against HA and NA. In this study, we developed an NA active site proximity assay (NASPA) for measuring NAI antibodies and applied it to evaluate NA antigenicity and profile anti-NA antibodies in human sera. The assay is based on the ability to detect the binding of a large ‘bulky’ NA active site inhibitor in the presence of anti-NA antibodies by using a small reporter substrate such as 4-methylumbelliferyl-*N*-acetyl-α-D-neuraminic acid (MUNANA)^44^. The assay is not affected by the presence of anti-HA antibodies and consequently, can be performed with the same reagents (viruses and ferret antisera) used to assess HA antigenicity. NASPA also can be easily automated as the procedure involves using a defined amount of NA and sequentially adding test sera, a large bulky NA active site inhibitor, and a small NA activity reporter substrate. The flexibility of the modular approach combined with the ease of automation, indicates NASPA can be used to efficiently collect NA antigenic data or coupled with a simple activity assay to profile complex anti-NA antibody responses in humans.

## Results

### NAI antibody measurements are substrate-dependent

**Fig. 1:**
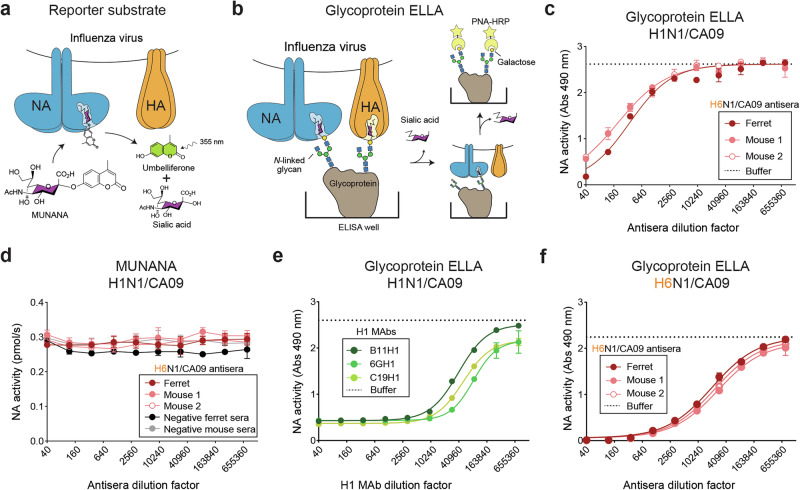
Large NA substrates are needed to detect steric NA inhibitory (NAI) antibodies. **a**, **b** Diagrams showing the detection of NA enzymatic activity with the (**a**) small monovalent reporter substrate MUNANA and (**b**) multivalent glycoproteins by ELLA. MUNANA cleavage is monitored by fluorescent detection of the released umbelliferone. Cleavage of the sialylated glycans on the glycoprotein is measured by HRP-conjugated peanut agglutinin (PNA-HRP) binding to the exposed terminal galactose residue. **c**, **d** NA activity inhibition by ferret and mouse antisera was measured with a H1N1/CA09 virus by (**c**) ELLA using the glycoprotein fetuin and (**d**) MUNANA. Ferret and mouse antisera were generated by intranasal infection with a reassortant virus (H6N1/CA09) carrying a mismatched HA (H6) and a matching NA (N1/CA09). **e** NA activity in the H1N1/CA09 virus was measured by ELLA in the presence of serially diluted H1 MAbs that inhibit the receptor binding function of the virus (Supplementary Table 1). Purified H1 MAbs were diluted as indicated from an initial concentration of ~1 mg/ml. **f** NA activity in the H6N1/CA09 virus was measured by ELLA in the presence of serially diluted ferret and mouse antisera raised against the H6N1/CA09 virus.

NA activity is often measured using small monovalent reporter substrates like MUNANA or more biologically relevant multivalent substrates such as the glycoprotein fetuin. With MUNANA (Fig. 1a), NA activity is readily measured by using fluorescence to quantify the 4-methyl umbelliferone that is released following sialic acid cleavage. In contrast, NA activity measurements with glycoproteins require more complex assays like ELLA, which monitor sialic acid cleavage by using peanut agglutinin binding to the exposed galactose residues on the glycoprotein (Fig. 1b). Initially, we used both assays to measure NA activity from the H1N1 strain A/California/07/2009 (H1N1/CA09) in the presence of ferret or mouse antisera that were raised against a reassortant virus (H6N1/CA09) carrying H6 and the NA from H1N1/CA09. With the fetuin-based ELLA, ferret and mouse antisera both reduced the NA activity by more than 50% (Fig. 1c), whereas no significant activity changes were observed with the smaller substrate MUNANA (Fig. 1d). This observation is in line with the common practice of using ELLA to measure serum NAI antibodies and several reports showing NAI antibodies can sterically hinder the ability of the NA active site to bind sialic acid residues on large *N-*linked glycans, but not on smaller reporter substrates like MUNANA^40,45–48^.

### HA contributes to the viral NA activity measurements with multivalent glycoprotein substrates

Bovine fetuin contains up to three sialylated *N-*linked glycans and several sialylated *O-*linked glycans^49^ that can act as substrates for NA or potential receptors for HA (Fig. 1b). Consequently, viruses can bind to fetuin via HA which localizes the neighboring NA proteins near the sialylated glycan substrates, likely increasing the apparent activity of NA in a virus^50,51^. To test the contribution of HA binding when fetuin is localized to a surface, we measured the NA activity in the H1N1/CA09 virus by ELLA in the presence of several H1 monoclonal antibodies (MAbs) that inhibit H1N1/CA09 receptor binding (Supplementary Table 1). All the H1 MAbs significantly reduced the NA activity measured by ELLA (Fig. 1e), resulting in false positive NAI titers of more than 10,000 (e.g., reciprocal dilution of the half-maximal inhibitory concentration (*IC*_*50*_) of the antibody). In line with this result, we also observed a significant NA activity decrease in the H6N1/CA09 virus when it was measured by ELLA in the presence of the H6N1/CA09 ferret and mouse antisera (Fig. 1f), which can also inhibit H6N1/CA09 receptor binding (Supplementary Table 1). With the H6N1/CA09 virus these antisera produced NAI titers of ~10,000 compared to ~160 with the HA subtype mismatched H1N1/CA09 virus. Together, these results illustrate how antibodies against HA, especially those that inhibit the receptor binding function, can produce false positive or inflated NAI antibody measurements by ELLA. Supporting this conclusion, several previous studies have also reported that ELLA is subject to interference by other HA antibodies^37–39^, making it difficult to establish NAI correlations^27^, or implement it for monitoring NA antigenic drift with the same reagents (viruses and ferret antisera) used for analyzing HA antigenic drift.

### Development and pilot testing of an NA active site proximity assay (NASPA)

**Fig. 2:**
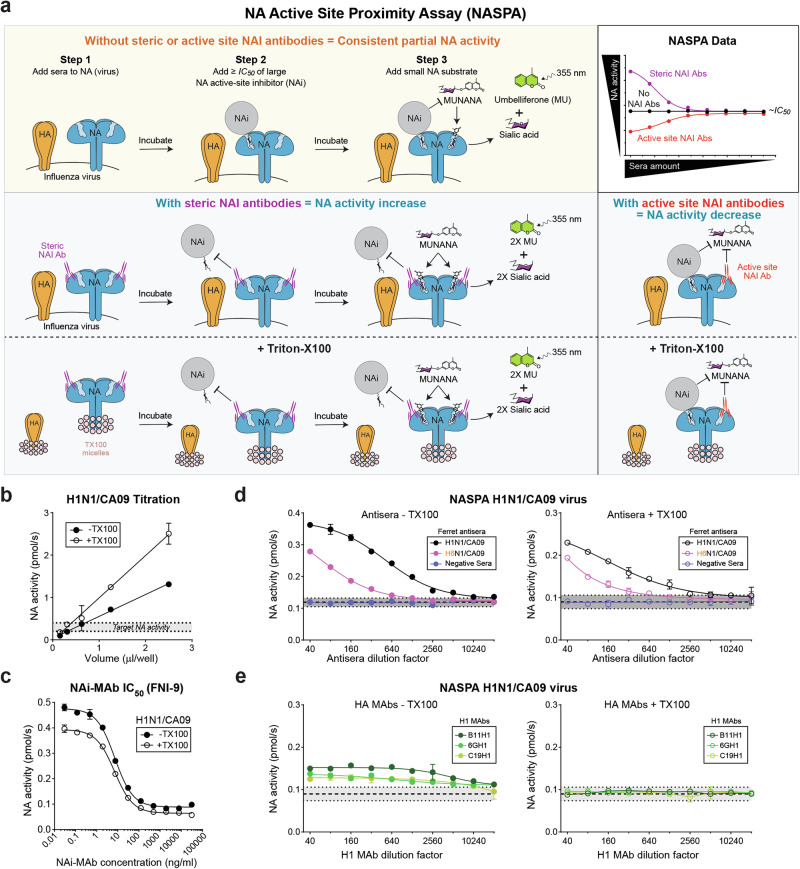
NASPA development and pilot testing. **a** Diagram of NASPA showing the expected outcomes of the three steps in the presence (blue region) or absence (orange region) of steric and active site binding NAI antibodies. Following (step 1) a sera titration with a fixed amount of intact or detergent treated virus, (step 2) a large ‘bulky’ NA inhibitor (NAi) is added at the half-maximal inhibitory concentration (*IC*_*50*_), and (Step 3) the ability of steric and active site NAI antibodies to block (steric) the large NA inhibitor or contribute to inhibition (active site) is detected by MUNANA. **b** H1N1/CA09 virus in the absence or presence of TX100 (0.025% final concentration) was serially diluted and NA activity was measured for 10 min after MUNANA addition. **c**. Inhibition curves are displayed for the NAi-MAb (FNI-9) with untreated and TX100-treated H1N1/CA09 virus. The NAi-MAb was incubated 3 h at 37 °C prior to measuring NA activity by MUNANA. **d**, **e** NA activity data is displayed from NASPA that was performed using untreated and TX100-treated H1N1/CA09 virus with the indicated (**d**) ferret antisera or (**e**) H1 MAbs that inhibit the receptor binding function of the H1N1/CA09 virus. Assays were performed in duplicate using NAi-MAb amounts approximating the *IC*_*80*_ and are displayed as the mean ± the standard deviation (SD). NA activities were read for 10 min after MUNANA addition. Gray regions indicate the mean (dashed line) ± 3 SDs (dotted line) that were determined using (**d**) negative control ferret sera or (**e**) multiple wells containing PBS.

An ideal NAI assay should be compatible with the different NAs from circulating viruses, capable of differentiating steric versus active site inhibition, and insensitive to the presence of HA antibodies as this would eliminate the need for NA specific reagents (e.g., strains or antisera). We posited that this could be achieved by creating a NA active site proximity assay (NASPA) that uses a large chemical NA active-site-binding inhibitor (NAi), or monoclonal antibody, to replace the function of the sialylated glycans on fetuin that serve as NA substrates in ELLA (Fig. 2a). We initially set the NAi at a half-maximal concentration (*IC*_*50*_) or higher to create NA populations with both bound and accessible active sites. Under these conditions, we hypothesized that sera containing steric NAI antibodies (Fig. 2a, step 1) would block the large NAi from binding the active site (Fig. 2a, step 2), resulting in NA activity increases that could be measured using the small substrate MUNANA (Fig. 2a, step 3). We speculated that these conditions would also enable detection of NAI antibodies that bind the active site as these could target the sites not occupied by the NAi, resulting in NA activity decreases that could be confirmed in a MUNANA only assay.

We initially tested this concept with the H1N1/CA09 virus and several recently identified NA inhibiting monoclonal antibodies (NAi-MAbs) FNI-9, FNI-19, and 1G01, which inhibit a broad range of NAs from type A and B viruses by binding to the active site^52,53^. The pilot test was also performed in the absence and presence of the detergent Triton X-100 (TX100) at a final concentration of 0.025% to compare results from intact virions to spatially separated HA and NA antigens (Fig. 2a). We first titrated the virus by measuring NA activity with MUNANA to identify amounts that cleaved ~0.25–0.5 pmol/sec of substrate (Fig. 2b). Next, we measured the IC_50_ of the NAi-MAbs (FNI-9, FNI-19 and 1G01) with and without TX100 using the standardized virus amounts (Fig. 2c and Supplementary Fig. 1a). We then incubated the same virus amounts with serial dilutions of ferret antisera raised against H1N1/CA09 or a H6N1/CA09 reassortant virus to investigate if the sera possess detectable steric or active site NAI antibodies by NASPA and the influence of anti-HA antibodies against the test virus. Each well was then incubated with the NAi-MAb at an inhibitory concentration greater than 50% prior to measuring NA activity with MUNANA. Using this approach, both ferret antisera increased the NA activities above the negative control ferret sera with all three NAi-MAbs and the activities decreased to control levels as the sera were diluted (Fig. 2d, Supplementary Fig. 1b, c).

We then determined the NASPA titers for each serum by identifying the largest dilution factor that showed NA activities more than 3 standard deviations (SDs) above the mean NA activity from the negative control sera. The results with all three NAi-MAbs showed that TX100 reduced the NASPA titers from the ferret antisera against H1N1/CA09 that possessed antibodies against the HA in the H1N1/CA09 test virus (Table 1). Importantly, TX100 treatment also eliminated false positive NA activity increases that were observed when NASPA was performed with the three MAbs that recognize the receptor binding domain of the HA in H1N1/CA09 (Fig. 2e). Together, these results suggested that NASPA could be used to measure NAI antibodies and that NA and HA should be separated to avoid potential interference from antibodies against HA.

**Table 1:** NASPA titers against H1N1/CA09 virus ± Triton X-100 with different NAi-MAbs

	NAi-MAb (FNI-9)		NAi-MAb (FNI-19)^a^		NAi-MAb (1G01)^a^	
	−TX100	+TX100	−TX100	+TX100	−TX100	+TX100
H1N1CA/09 Ferret antisera	10240	2560	5120	1280	5120	1280
H6N1CA/09 Ferret antisera	640	640	320	320	320	320
Negative Ferret Sera	<40	<40	<40	<40	<40	<40

NASPA titers correspond to the highest dilution factor 3 SD above the negative ferret sera mean.

^a^Raw data are shown in Supplementary Fig. 1.

### Compatibility and optimization of NASPA with the different NAs from influenza vaccines

**Fig. 3:**
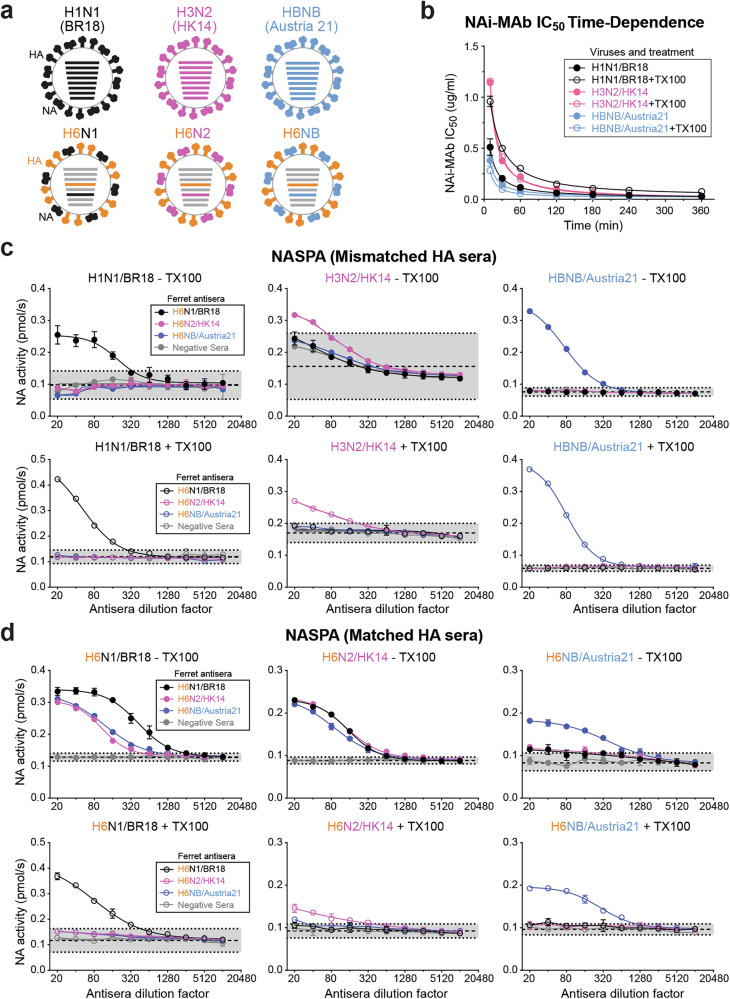
NASPA vaccine strain testing with HA mismatched and matched ferret antisera. **a** Schematic of the type A (H1N1 and H3N2) and B (HBNB) vaccine strains and the corresponding H6 reassortant viruses that were analyzed by NASPA. **b** The NAi-MAb (FNI-9) *IC*_*50*_ was measured after different incubation times with the indicated vaccine viruses in the absence and presence of TX100. Results are displayed as the mean ± SD from two independent experiments. **c** NASPA results are displayed for the three vaccine strains in the absence (upper panels) or presence of TX100 (lower panels) and the indicated ferret antisera raised against H6 reassortant viruses carrying the vaccine strain NAs. Negative ferret serum was included as a control. **d** NASPA results are shown for the H6 reassortant viruses carrying the vaccine strain NAs with the ferret antisera that were raised against the same viruses. NASPA was performed in the absence (upper panels) or presence of TX100 (lower panels). Negative ferret serum was included as a control. All NASPA assays were performed in duplicate using NAi-MAb concentrations corresponding to the *IC*_*80*_ and are shown as the mean ± SD of the NA activities measured for 10 min after MUNANA addition. Gray regions indicate the mean (dashed line) ± 3 SDs (dotted line) from the negative control sera.

Vaccines against circulating influenza viruses generally include two type A subtypes (H1N1 and H3N2) and a type B virus that is currently from the Victoria lineage. Therefore, we performed a more thorough analysis of NASPA using different vaccine strains and the NAi-MAb FNI-9 that possesses broad NA binding properties^52^. For the vaccine strains we used H1N1 (A/Brisbane/02/2018), H3N2 (A/Hong Kong/4801/2014), and a type B (B/Austria/1359417/2021) virus, as well as H6 reassortant viruses that carried NAs from the same vaccine strains (Fig. 3a).

After titrating the vaccine viruses, we measured the *IC*_*50*_ of the NAi-MAb for each virus after different incubation times in the absence and presence of TX100 (Supplementary Fig. 2). A temporal plot of this data showed that the NAi-MAb binding required ~180 min at 37 °C to reach equilibrium for all three NAs (Fig. 3b). We did note some NA-dependent variation in the equilibration times and TX100 effects. Next, we tested different ferret sera incubation times and observed consistent results with each vaccine virus after 30 min (Supplementary Fig. 3). Finally, we used these parameters to analyze each vaccine virus in the absence and presence of TX100 with a panel of NA ferret antisera that were generated using H6 reassortant viruses to avoid recognition of HA in the vaccine strains (Fig. 3c). The H1N1 and the type B vaccine viruses were only recognized by matching NA ferret antiserum and the data did not change when TX100 was present (Fig. 3c, left and right panels). Unexpectedly, all three NA ferret antisera and the control sera caused NA activity increases with the H3N2 vaccine virus without TX100 (Fig. 3c, upper middle panel). TX100 addition minimized these nonspecific interactions (Fig. 3c, lower middle panel), suggesting that binding of the H3N2 virus to serum components, presumably via HA, may block the access of the NAi-MAb to the NA in the H3N2 virus.

### Minimizing anti-HA antibody interference during NASPA

To examine how NASPA performs with different NAs in the presence of anti-HA antibodies, we tested the H6 reassortant viruses carrying the vaccine strain NAs (Fig. 3a) with the strain specific ferret antisera that were generated using these same viruses. In the absence of TX100, all three HA matched ferret antisera showed NA activity increases that varied with each virus (Fig. 3d, upper panels), likely due to the presence of antibodies against the HA (e.g., H6) in the reassortant viruses. However, when TX100 was present only the ferret antisera raised against the test viruses blocked the NAi-MAb and significantly increased NA activity (Fig. 3d, lower panels), indicating that anti-HA antibodies can likely interfere with the NAi-MAb binding to NA in an intact virus. Following this observation, we titrated the TX100 amount with the same viruses and found that final concentrations between 0.025–0.1% TX100, or 2–10 times the TX100 critical micelle concentration (CMC), prevented the anti-H6 antibody interference and produced similar results (Supplementary Fig. 4). We then applied these parameters and obtained similar NASPA results with viral and recombinant NAs (Supplementary Fig. 5), further supporting that these conditions, including detergent, limit potential interference from anti-HA antibodies.

### Optimization of NAi-MAb concentrations, NA amounts, and substrate incubation times

NASPA titers are dependent on the ability of antibodies in sera to block the binding of the NAi-MAb. Therefore, we measured NASPA titers for the three vaccine NAs in the presence of increasing NAi-MAb concentrations to determine the functional range for the inhibitor. Although the minimal NAi-MAb concentrations needed to obtain a NASPA titer varied between the NAs, we did obtain consistent NASPA titers for all three NAs when the NAi-MAb amounts were above the *IC*_*50*_ (Supplementary Fig. 6), indicating NASPA titers are relatively insensitive to changes in the NAi-MAb concentrations above the *IC*_*50*_.

Lowering the NA amounts in NASPA can potentially increase the sensitivity for samples with low anti-NA antibody quantities. Therefore, we measured NASPA titers with decreasing amounts of the three vaccine NAs after different MUNANA incubation times. These included kinetic measurements for 10 min after MUNANA addition and endpoint measurements 10 min and 2 h after MUNANA addition (Supplementary Fig. 7). As expected, NASPA titers from the kinetic and 10 min endpoint readings were similar, and the ability to detect titers was lost with the lower NA amounts (Supplementary Fig. 7d–f). At the 2 h endpoint, NASPA titers were more consistent across a wide range of NA amounts (e.g., 4–16 fold differences), indicating longer substrate incubation times with lower NA amounts can increase the consistency and sensitivity of NASPA results.

### NASPA and ELLA comparison using MAbs against N1, N2, and type B NA

**Fig. 4:**
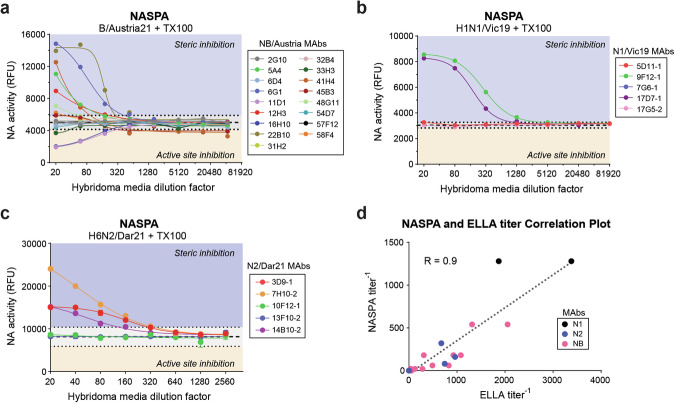
NASPA and ELLA results from MAbs against N1, N2, and type B NA correlate. NASPA data obtained with TX100-treated (**a**) B/Austria21, (**b**) H1N1/Vic19, and (**c**) H6N2/Dar21 vaccine strains and the indicated MAb hybridoma medium are displayed. Medium from duplicate hybridoma clones was analyzed, and a single representative of each is shown. The type B and H1N1 virus analysis used the NAI MAb FNI-9 at an ~*IC*_*80*_, whereas the H3N2 virus analysis used a modified NAi-MAb (FNI-9 R27D) at an ~*IC*_*80*_. Endpoint NA activities (RFU) were measured after a 10 min incubation with MUNANA at 37 °C. Gray regions indicate the mean (dashed line) ± 3 SDs (dotted line) from control reactions containing serially diluted hybridoma medium. **d** Correlation plot of NASPA and ELLA titers obtained with the same viruses and MAb hybridoma media. Pearson’s correlation coefficient (R) for the linear regression (dotted line) of the 27 MAb hybridoma media is shown. Negative (active site binding) NASPA titers were treated as positive for the correlation, and values below the limit of detection were assigned 0.

Since NAI titers are commonly measured with a fetuin-based ELLA approach^40,41,54^, we compared NASPA and ELLA titers with a panel of MAbs in hybridoma media that we validated by ELISA for binding against N1, N2, and type B NA from recent vaccine strains (Supplementary Figure 8 and Table 2). Contrary to ELLA, which only shows NA activity decreases (Supplementary Fig. 8d–f), NASPA displayed activity increases and decreases with the MAbs against the type B NA (Fig. 4a). We attributed the expected activity increases to steric inhibition of the NAi-MAb, and the activity decreases suggested NASPA may also recognize MAbs that bind the active site and inhibit NA because it uses NAi-MAb amounts in the *IC*_*50*_*-IC*_*80*_ range. Supporting this conclusion, each MAb that showed decreased activity in NASPA also reduced the NA activity measured by MUNANA alone (Supplementary Fig. 10). With the MAbs against N1, two out of the five gave positive NASPA titers (Fig. 4b). For the NA from the H3N2 vaccine strain A/Darwin/9/2021 (Dar21), we did not identify an *IC*_*50*_ for the NAi-MAb (FNI-9) as it was above 4 mg/ml (Supplementary Fig. 9).

**Table 2:** NASPA, ELLA and ELISA titers of MAbs against type B NA (NB), N1 and N2

Assay Virus	MAb	ELISA titer^a^	ELLA titer^b^	NASPA titer^c^
NB/Austria21 Virus	6G1-1	640	1315	540
	22B10-1	>1280	2055	540
	11D1-1	640	925	−180*
	12H3-1	320	310	180
	16H10-1	640	1080	−180*
	5A4-1	320	490	60
	41H4-1	160	830	60
	45B3-1	320	280	20
	48G11-1	320	125	20
	58F4-1	80	40	20
	33H3-1	80	35	−20*
	2G10-1	320	50	<20*
	31H2-1	80	55	<20
	6D4-1	160	35	<20
	32B4-1	80	<20	<20
	54D7-1	40	<20	<20
	57F12-1	40	<20	<20
N1/Vic19 Virus	9F12-1	512	3380	1280
	17D7-1	128	1870	1280
	7G6-2	512	<20	<20
	17G5-2	512	<20	<20
	5D11-1	128	<20	<20
N2/Dar21 virus	7H10-2	160	675	320**
	3D9-1	640	960	160*
	14B10-2	320	745	80*
	13F10-2	>1280	<20	<20*
	10F12-1	320	<20	<20

^a^End point titers correspond to the lowest dilution 2-fold above the control (Supplementary Fig. 8a–c).

^b^ELLA titers correspond to the IC_50_ (Supplementary Fig. 8d–f).

^c^NASPA titers are the highest dilution factor above or below (negative values) the mean control ±3 SD.

Displayed enzymatic (*) inhibition or (**) activation with MUNANA (Supplementary Fig. 9).

We overcame this problem by introducing a structure-guided substitution (R27D in the FNI-9 light chain). This substitution increased the NAi-MAb binding affinity by more than 10-fold (Supplementary Fig. 9), likely by complementing the charge of residue 344 K in Dar21 that was commonly a negatively charged residue (E) in NAs from earlier H3N2 strains. With this modified NAi-MAb we observed positive NASPA titers with three of the five N2 MAbs (Fig. 4c). We then plotted the NASPA titers versus the ELLA titers and observed a good linear correlation (R = 0.9), although the NASPA titers were generally lower (Fig. 4d and Table 2). We also noted that 24 out of the 27 MAbs showed the same positive and negative results and that the three which differed gave low ELLA titers (Fig. 4a and Table 2). Together, these results show that NASPA provides similar data with individual antibodies as ELLA with the added advantage that it can also differentiate steric and active site-binding NAI antibodies in a shorter amount of time. While the need to modify the NAi-MAb upon rare evolutionary changes to the active site can introduce a reagent lag time, any need for an update is likely to reflect an antigenic change in NA.

### NASPA can provide antigenic data for NAs from type A H1N1 and type B strains

**Fig. 5:**
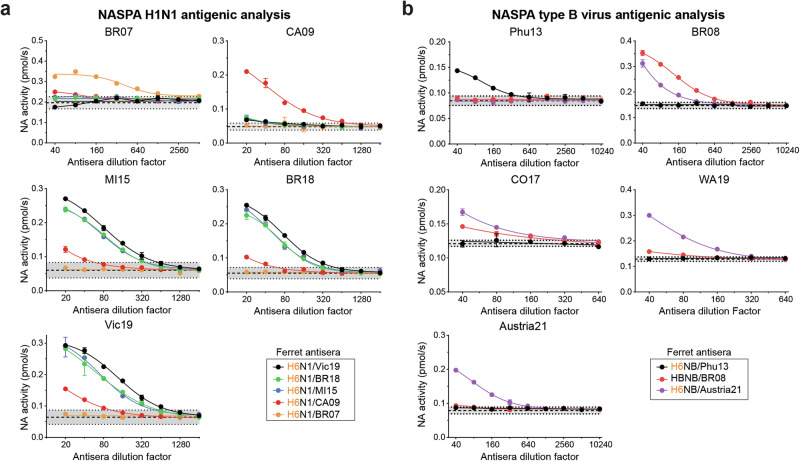
Antigenic analysis of NAs from recent H1N1 and type B vaccine strains by NASPA. **a** NASPA results for each TX100-treated H1N1 vaccine strain with ferret antisera raised against the indicated reassortant virus are shown. The H1N1 strains A/Brisbane/59/2007 (BR07), A/California/07/2009 (CA09), A/Michigan/45/2015 (MI15), A/Brisbane/02/2018 (BR18) and A/Victoria/2570/2019 (Vic19) were analyzed. **b** NASPA results for each TX100-treated type B vaccine strain with ferret antisera raised against the indicated wild type and reassortant virus are displayed. The B Yamagata lineage strain B/Phuket/3073/2013 (Phu13) and the B Victoria lineage strains B/Brisbane/60/2008 (BR08), B/Colorado/06/2017 (CO17), B/Washington/02/2019 (WA19) and B/Austria/1359417/2021 (Austria21) were tested. All analysis were performed in duplicate using NAi-MAb concentrations corresponding to the *IC*_*80*_ and are shown as the mean ± SD of the NA activities measured for 10 min after MUNANA addition. Gray regions indicate the mean (dashed line) ± 3 SDs (dotted line) of the control wells.

Monitoring NA antigenic drift in circulating strains is an ideal application for NASPA as it is insensitive to the presence of antibodies against HA. We tested this possibility using a panel of recent H1N1 vaccine strains from 2007 to 2019 which contained several strains we previously analyzed by ELLA^35^. After titrating the viruses with MUNANA and determining the NAi-MAb *IC*_*50*_ for each strain (Supplementary Fig. 11a), we set up plates to individually analyze each strain with the five ferret antisera raised against H6 reassortant viruses carrying the same NAs. The NASPA graphs indicated that the NA from the H1N1 strain A/Brisbane/59/2007 (BR07) is antigenically distinct from the NAs of post-pandemic H1N1s with slight similarity to the NA from the original 2009 H1N1 pandemic strain CA09 (Fig. 5a). In contrast, NAs from the H1N1 strains A/Michigan/45/2015 (MI15), A/Brisbane/02/2018 (BR18) and A/Victoria/2570/2019 (Vic19) all showed high antigenic similarity that was slightly related to the NA from H1N1/CA09. This conclusion was supported by the NASPA titers which showed the same trends as the previously reported ELLA titers (Table 3 and ref. ^35^).

**Table 3:**
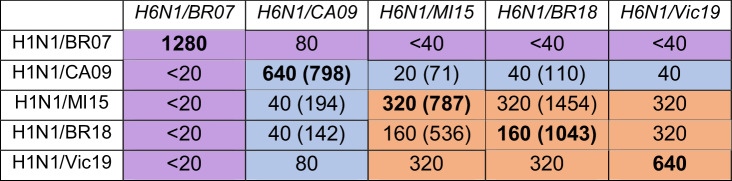
NASPA and previously determined ELLA titers for H1N1 vaccine viruses

NASPA titers for each virus were generated using the indicated ferret antisera (italics) and correspond to the highest dilution factor above the control mean + 3 SD. Titers for NAs matching the antisera are bold. ELLA titers in parenthesis are from a previous study using the same ferret sera and viruses^35^. Antigenically similar, related, and distinct NAs based on two-way test results are highlighted in orange, light blue, and purple, respectively.

NAs from type B vaccine strains have received little attention due to the difficulties in generating antisera that do not recognize HA in the test virus. Therefore, we analyzed several type B vaccine strains using ferret antisera raised against viruses that carried three of the type B NAs. After titrating and determining the NAi-MAb *IC*_*50*_ for each virus (Supplementary Fig. 11b), we performed the analysis. The NASPA results showed that the NA in the Yamagata-lineage strain B/Phuket/3073/2013 (Phu13) is antigenically distinct from the four Victoria-lineage strains (Fig. 5b and Table 4). Within the Victoria lineage, results with B/Brisbane/60/2008 (BR08) antisera showed progressively lower reactivity by year of virus isolation, indicating that the NA antigenicity has incrementally changed from BR08-like to B/Austria/1359417/2021-like (Austria21). However, the incremental change was less apparent with the Austria21 antisera. These observations suggest that the cumulative changes near the active site of the NAs from the Victoria lineage led to the recognition of a new dominant epitope that is more cross reactive with earlier strains, and we speculate that the switching of dominant epitopes may contribute to NA evolution in type B viruses.

**Table 4:**
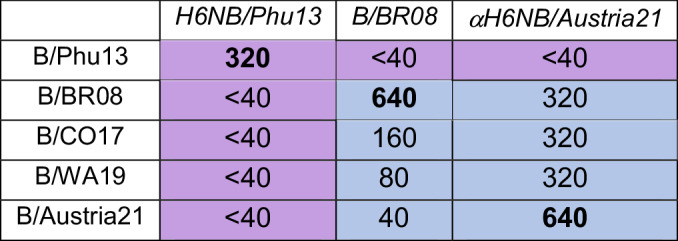
NASPA titers of type B vaccine viruses

NASPA titers for each virus were generated using the indicated ferret antisera (italics) and correspond to the highest dilution factor above the control mean + 3 SD. Titers for NAs matching the antisera are bold.

Antigenically related and distinct NAs are highlighted in light blue and purple, respectively.

### Adult human sera commonly possess steric and active site-binding NAI antibodies

**Fig. 6:**
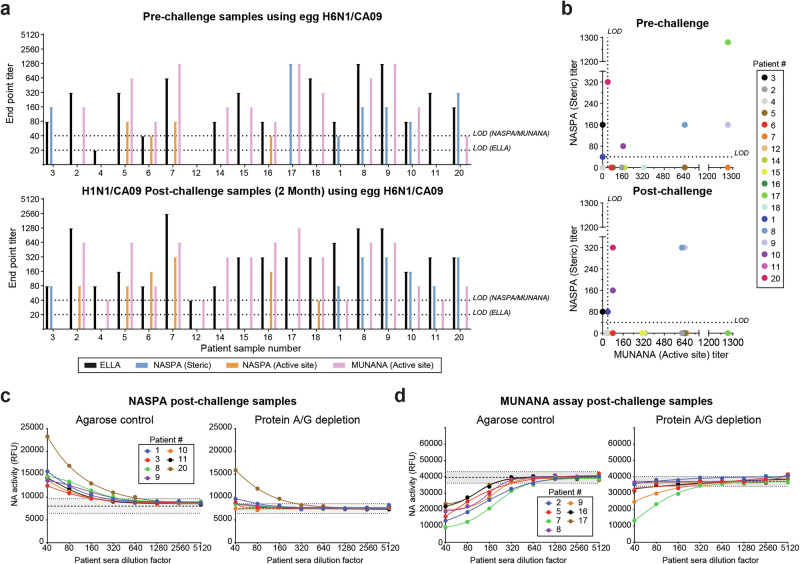
Analysis of adult NAI antibody responses pre- and post-challenge with H1N1/CA09 virus. **a** Endpoint titers determined with NASPA and a MUNANA activity assay are displayed for the indicated patients pre-challenge (upper panel) and 2 months post-challenge (lower panel) along with the ELLA titers previously reported for these samples^28^. Data was obtained using a TX100-treated H6 reassortant virus grown in eggs that contained the NA from the H1N1/CA09 challenge strain **b** Pre-challenge (upper panel) and post-challenge (lower panel) correlation plots showing the steric and active site NAI endpoint titers that were obtained from each serum with NASPA and MUNANA, respectively. Limits of detections (LODs) are shown by dotted lines. Overlapping dots were offset, and values below the LOD were assigned 0. **c**, **d** Post-challenge sera that showed strong (**c**) steric or (**d**) active site NAI antibody responses were depleted of immunoglobulin with protein A and G agarose or control agarose beads prior to performing NASPA or MUNANA analyses. Results are from one of two independent experiments. Assays were run with TX100-treated H6 reassortant virus using NAi-MAb concentrations corresponding to the *IC*_*80*_. NA endpoint activities were measured 2 h after MUNANA addition. Gray regions indicate the mean (dashed line) ± 3 SDs (dotted line) of the control wells.

NASPA can theoretically be coupled with a simple activity assay like MUNANA to effectively measure steric and active site-binding NAI antibody responses in human sera. As a pilot test, we obtained previously analyzed sera samples from an H1N1 human challenge study^28^ and performed NASPA and a MUNANA analysis in a blinded fashion using the H1N1 challenge virus and a H6 reassortant carrying the same NA. With both test viruses steric and active site NAI antibodies were readily detected, and the latter was confirmed to be even more prevalent by the MUNANA analysis (Fig. 6a and Supplementary Fig. 12). Unblinding the results revealed that the combined steric and active site NAI titers observed with MUNANA aligned well with the previously obtained ELLA titers^28^. Interestingly, the combined results also showed that some individuals possess either steric or active site NAI antibodies whereas others possessed both and these profiles showed some changes post-challenge (Fig. 6a, b).

The prevalence of active site NAI antibodies was quite unexpected. Therefore, we questioned if the activity of this labile and Ca^2+^ sensitive enzyme^2^ was reduced by other factors than antibody binding. To examine this possibility, we depleted the immunoglobulin from the strongest post-challenge sera samples using a combination of protein A and G agarose and repeated the NASPA and MUNANA analyses (Fig. 6c, d). The activity changes measured by NASPA and MUNANA in all cases were reduced following protein A and G incubation, and in most they were completely lost, confirming the results were largely antibody-mediated. We also noted that some sera showed reduced signals after incubation with the control agarose beads likely due to nonspecific binding. These results indicate that NAI antibody responses in adults are complex and function by two different mechanisms of NA inhibition that can be differentiated by coupling NASPA with a simple MUNANA activity assay.

### NASPA can utilize large “bulky” synthetic NA inhibitors

**Fig. 7:**
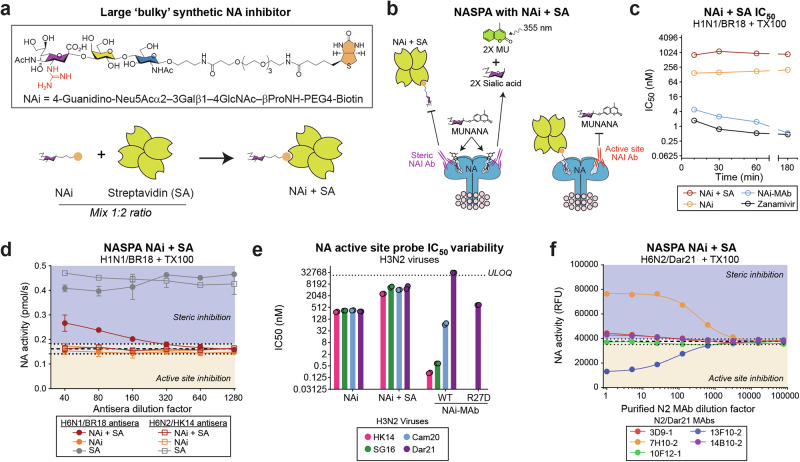
NASPA is compatible with a bulky synthetic NA inhibitor. **a** Diagram showing the novel chemoenzymatically synthesized biotinylated NA inhibitor (NAi: 4-guanidino-Neu5Acα2–3 Galβ1–4GlcNAc–βProNH-PEG4-Biotin) that was combined with streptavidin (SA) for use in NASPA. **b** NASPA schematic showing how the synthetic NA inhibitor bound to streptavidin (NAi + SA) can detect steric (purple) or active site (red) NAI antibodies. **c** *IC*_*50*_ values are displayed for the NAi, NAi + SA, NAi-MAb (FNI-9), and zanamivir after the indicated incubation times with TX100-treated H1N1/BR18 virus. **d** Streptavidin binding to the NAi is required for NASPA. NA activity data is displayed from NASPA performed using TX100-treated H1N1/BR18 virus with the NAi, NAi + SA, or SA and the indicated ferret antisera. Assays were performed in duplicate using amounts approximating the *IC*_*65*_ and are displayed as the mean ± SD. Gray region indicates the mean (dashed line) ± 3 SDs (dotted line) of the mock control wells. **e** *IC*_*50*_ values of the NAi, NAi + SA, and the NAi-MAbs (FNI-9 and FNI-9 R27D) for the indicated H3N2 vaccine strains are displayed. Each assay was run twice using either a 30 min incubation (NAi and NAi + SA) or 3 h incubation (NAi-MAbs) at 37 °C. **f** Representative NA activity data set is shown from NASPA that was performed using TX100-treated H3N2/Dar21 virus with the NAi + SA mixture and the indicated purified N2 MAbs. Purified N2 MAbs were diluted as indicated from a starting concentration of ~20 μg/ml. The assay was performed using NAi + SA amounts approximating the *IC*_*80*_. Gray region indicates the mean (dashed line) ± 3 SDs (dotted line) of the mock control wells.

Although NASPA with the NAi-MAb provided reproducible results in a time efficient manner, we sought to improve it further by replacing the NAi-MAb with a large bulky synthetic inhibitor which can potentially bind different NAs with similar affinities. For this analysis, we took advantage of a recently synthesized biotinylated sialoside-based NA inhibitor (4-guanidino-Neu5Acα2–3 Galβ1–4GlcNAc–βProNH-PEG4-Biotin) that was shown to bind NAs with nanomolar affinity^55^ and was also inhibited by ferret antisera in an ELISA format (Supplementary Fig. 13). Based on these results, we reasoned that the synthesized NAi size could be increased by mixing it with streptavidin (SA) and the mixture could be used for NASPA (Fig. 7a, b). After mixing with streptavidin (1:2 molar ratio, NAi + SA) the *IC*_*50*_ of the synthesized NAi for N1 slightly increased and the equilibrium was reached within 10–30 min (Fig. 7c and Supplementary Fig. 14a), compared to ~3 h for the NAi-MAb. We then tested the compound in NASPA using the H1N1/BR18 virus and only observed NA activity increases with the ferret antisera raised against H6N1/BR18 and the NAi + SA mixture (Fig. 7d). Somewhat unexpected, the NASPA titer of this ferret antisera with the NAi + SA mixture (~160) was identical to the titer achieved with the NAi-MAb (Table 3), indicating the biotinylated sialoside-streptavidin complex is suitable for NASPA.

We previously modified the NAi-MAb (FNI-9) to improve the affinity for the NA from a recent H3N2 virus, so we measured the *IC*_*50*_ of the synthesized NAi ± SA for several recent H3N2 viruses. In contrast to the NAi-MAb (FNI-9), both the NAi and NAi + SA mixture showed consistent *IC*_*50*_ values suggesting it could be used at a set concentration for NASPA (Fig. 7e). Finally, we tested the synthetic NAi using purified N2 MAbs. With the NAi + SA mixture, steric inhibition was observed with three of the purified MAbs, and another showed an activity decrease indicative of active site binding. As these results were somewhat different than those from the hybridoma media with unknown antibody concentrations (Fig. 4c), we also analyzed the purified N2 MAbs using NASPA with modified NAi-MAb and observed a similar pattern (Supplementary Fig. 14b). We did note the activity increases from two of the MAbs were more pronounced, likely due to the different spatial footprints of the NAi + SA and the NAi-MAb. Interestingly, the active site binding N2 MAb (13F10), which was confirmed with a MUNANA assay (Supplementary Fig. 14c), could potentially serve as an effective NAi-MAb for NAs from recent H3N2 viruses. Altogether, these data demonstrate that NASPA is an efficient and cost-effective platform that can utilize different large ‘bulky’ NA active site inhibitors to profile NA antigenicity and NAI antibody responses, making it well suited to aid the development of new influenza vaccines containing NA.

## Discussion

Currently influenza vaccine strains are recommended based on the HA antigens due to the absence of a simple NA antigenic assay that can be implemented to help select appropriate vaccine strains for the NA antigens. In this study, we describe the development of a cost-effective NA active site proximity assay (NASPA) for measuring NAI antibody responses that can be used to monitor changes in NA antigenicity. The assay is performed in a 96-well plate format and is based on the concept of replacing the large sialylated glycans that are used for NA substrates in ELLA with a large “bulky” NA inhibitor (NAi). The entire assay can be run in a single day, and it only requires two up front titrations steps to determine the NA and inhibitor amounts prior to assay execution which involves sequentially adding sera, inhibitor, and substrate to the predetermined NA amount from viruses or recombinant protein. We also demonstrated some applications of this automatable assay by profiling the antigenicity of NAs from different vaccine strains and differentiating the steric and active site NAI antibody responses in human sera by coupling it with a simple activity assay.

A major advantage of NASPA is that it is insensitive to the presence of antibodies against the HA in the test virus, making it ideal for utilizing the same reagents (field strains and ferret antisera) for monitoring HA antigenicity in circulating strains to also analyze NA antigenicity. This was achieved by including detergent to separate the NA and HA antigens in the test virus and using a monovalent small molecule NA reporter substrate (MUNANA) for detection. These two steps eliminate potential steric interference from anti-HA antibodies^37–39^ as well as HA-mediated increases in the apparent activity of NA in a virus^50^ that can contribute to false positive and inflated NAI titer measurements by ELLA. This property is one of the reasons we used NASPA to examine NA antigenicity in type B viruses as it is difficult to develop reagents for these viruses that minimize potential interference from anti-HA antibodies. While we only analyzed a few strains, NASPA can easily be expanded to encompass more strains and ferret antisera.

We also examined NA antigenicity in recent H1N1 vaccine strains to demonstrate that the results are comparable to a previous ELLA analysis^35^, but we did not analyze recent H3N2 vaccine strains due to the poor and variable NAi-MAb binding. We did address this issue for a recent H3N2 vaccine strain by introducing structure assisted modifications into the NAi-MAb. In addition, we also overcame this issue by generating a chemoenzymatically synthesized NAi that could be coupled with streptavidin. The incubation time for this NAi is ~2.5 h less than the NAi-MAb, and it showed similar binding affinities across the NAs we tested, potentially shortening the assay time by eliminating the ~3 h *IC*_*50*_ test for each NA. This demonstrated that there are multiple ways to adapt the assay for evolutionary changes in the NA, but NAs should likely use the same probe to avoid differences related to the footprint. Based on these beneficial attributes we are currently pursuing a large NA antigenic analysis of circulating viruses using both inhibitor approaches to establish a framework that can be implemented to help select vaccine strains for the NA antigens.

The surprising finding of prevalent active site-binding NAI antibodies in the human sera analyses led us to perform an additional immunoglobulin depletion step to ensure that the inhibition was indeed antibody-mediated. This confirmed the unexpected observation, suggesting that adults commonly possess antibodies capable of inhibiting substrate binding or reducing the activity of NAs in circulating strains. Supporting this conclusion, 6 out of the 7 individuals we observed with steric NAI antibodies post-challenge also possessed active site NAI antibodies, indicating that adults can generate polyclonal NAI responses that function by two different mechanisms. While this was confirmed by coupling NASPA with a simple activity assay, NASPA as described is performed with an NAi between the *IC*_*50*_*-IC*_*80*_, which enables detection of active site-binding NAI antibodies by an observed decrease in activity. The NAi concentration can easily be adjusted to bias for measuring steric NAI antibodies or both steric and active site binding. However, we recommend coupling it with a simple activity assay to confirm that steric and active site binding antibodies are not counter acting each other in the assay.

Since the challenge was performed in 2013–2014, several years after H1N1/CA09 had entered the population, it is possible that the NAI antibody response pattern we observed was due to more than one H1N1/CA09-like virus exposure. In line with this possibility, we isolated multiple NA MAbs that bound the NA active site from mice that had been immunized three times. Based on these results, we speculate that pediatric populations are likely to be more biased toward steric NAI antibodies, similar to what is observed in naïve animal models, and that active site NAI antibodies develop over time from multiple influenza infections. However, it is not clear how these patterns would change with NA antigenic drift. Regardless, distinguishing between steric and active site NAI antibody responses in humans may be critical for optimizing the design and administration of NA-based vaccines and for identifying a correlate of protection related to NA responses.

Mechanistically, anti-NA antibodies can provide protection by binding the active site (like FNI-9 and 1G01), sterically inhibiting the active site, activating antibody-dependent cellular cytotoxicity and phagocytosis *via* the Fcγ receptor, or a combination of these^45,47,48,52,53,56,57^. While NASPA is designed to detect steric and active site NAI antibodies, many of which likely activate the Fcγ receptor, it will likely not detect NA antibodies that bind epitopes away from the active site. However, this deficiency can potentially be used for screening antibodies that target regions away from the active site to determine if these types of antibodies are protective. Overall, we have established a time and cost-effective method that can be used to distinguish between steric and active site NAI responses in animals and humans, alleviate confounding results observed from ELLA due to anti-HA antibody interference, and help to select NA seasonal vaccine strains based on antigenic data.

## Materials and methods

### Viruses

Vaccine strains obtained from the WHO were propagated in 10-day-old specific pathogen-free (SPF) embryonated chicken eggs for 3 days at 33 °C. These included H1N1 strains: A/Brisbane/59/2007 (BR07), A/California/07/2009 (CA09), A/Michigan/45/2015 (MI15), A/Brisbane/02/2018 (BR18) and A/Victoria/2570/2019 (Vic19); H3N2 strains: A/Hong Kong/4801/2014 (HK14), A/Singapore/INFIMH-16-0019/2016 (SG16), A/Cambodia/e0826360/2020 (Cam20), and A/Darwin/9/2021 (Dar21); and influenza B strains from the Yamagata lineage (B/Phuket/3073/2013 (Phu13)) and the Victoria lineage strains: B/Brisbane/60/2008 (BR08), B/Colorado/06/2017 (CO17), B/Washington/02/2019 (WA19) and B/Austria/1359417/2021 (Austria21). The genetic composition of the reassortant viruses containing HA from the H6N2 strain A/turkey/Massachusetts/3740/1965 and the NA from the indicated vaccine strain and the six internal gene segments from the PR8 strain A/PR/8/1934 are provided in Supplementary Table 2. The viruses were generated by reverse genetics as described previously^58,59^ and propagated in SPF eggs and are referred to as: H6N1/BR07, H6N1/CA09, H6N1/MI15, H6N1/BR18, H6N2/HK14, H6N2/Dar21, H6NB/Austria21, and H6NB/Phu13. NAs in the reassortant viruses for the two B vaccine strains were chimeras that were generated by replacing the coding region for residues 36–437 in the N1/BR07 reverse genetics plasmid with the coding region for residues 34–466 of the indicated B vaccine strains.

### Animal ethics statement

All animal experiments were approved by the U.S. FDA Institutional Animal Care and Use Committee (IACUC) under Protocols #2001-05 and #2003-18. The animal care and use protocol meets National Institutes of Health (NIH) guidelines.

### Generation of ferret and mouse antisera

Antisera was obtained using intranasal inoculations. Inoculations were performed on 14-week-old male ferrets using allantoic fluid containing the indicated reassortant virus at an infectious titer of 10^6^–10^8^ TCID_50_/ml on MDCK cells. The inoculum (~0.5 ml) was equally dispensed between each nostril using a 1 ml syringe equipped with a 20 Gauge feeding needle. Body temperatures and weight were recorded daily for two weeks. Ferrets were anesthetized with an intramuscular injection of a ketamine/dexmedetomidine mix (5–8 mg/kg /40–50 μg /kg, respectively) and exsanguinated by cardiac puncture 3-week post-infection and ferret vital functions were observed for 10 min after the procedure. Sera samples were processed, aliquoted and stored at −80 °C. Mouse antisera were generated by inoculating anesthetized 8-week-old female DBA/2 J mice with allantoic fluid (~0.1 ml) containing the indicated reassortant virus diluted in PBS pH 7.2 to an infectious titer of 10^2^–10^4^ TCID_50_/ml on MDCK cells. Body temperatures and body weight were recorded daily for two weeks. Mice were anesthetized in an induction box via inhalation of 1–5% vaporized isoflurane in 100% oxygen and exsanguinated by cardiac puncture 3-week post-infection. Anesthesia was maintained at 2% isoflurane with a nose cone, and vital signs were monitored for 10 min after the procedure. Sera were processed, aliquoted, and stored at −80 °C.

### Enzyme-linked lectin assay (ELLA) titer determination

ELLA was performed on 96-well Maxisorp plates coated with 2.5 µg/well of bovine fetuin as previously described^40,41^. Test virus concentrations were first determined by adding 2-fold serial dilutions of virus in ELLA buffer 2-*N*-morpholino-ethanesulfonic acid (MES) 25 mM pH 6.5, 150 mM NaCl, 20 mM CaCl_2_, 1% bovine serum albumin) containing 0.5% Tween 20 to the fetuin-coated plates and incubating at 37 °C overnight. Plates were washed with 3 × 200 µl/well PBS pH 7.4 containing 0.05% Tween 20 (PBST), and 100 µl/well of horse radish peroxidase-linked peanut agglutinin diluted in ELLA buffer (~1 µg/ml) was added. Plates were incubated 2 h at room temperature, washed 3 × 200 µl/well with PBST, and OPD substrate was added. After a 10 min incubation at 37 °C, reactions were stopped with 100 µl/well 1 N sulfuric acid, and absorbance (Abs) at 490 nm was measured on a Cytation 5 (Biotek) plate reader. Test virus dilutions with an Abs 490 nm ~2.0 were used for the following assay. Antisera or antibodies were serially diluted in ELLA buffer containing 0.5% Tween 20, transferred (50 µl/well) to a fetuin-coated plate, and the test virus diluted in the same buffer was added (50 µl/well). Plates were incubated at 37 °C overnight and processed as described above. Abs 490 nm results were plotted with respect to antisera/antibody dilution factor in GraphPad Prism 8.0, and ELLA titers corresponding to the half-maximal inhibitory concentration (IC_50_) were determined by a variable slope four-parameter least squares regression analysis.

### Recombinant NAi-MAb expression

NAi-MAbs were all produced as recombinant murine IgG1 chimeras by transfection of XPi CHO cells and purification by protein A Sepharose (GenScript). Briefly, variable regions of the heavy- (V_H_) and light chain (V_L_) from the reported human antibodies FNI-9, FNI-19, 1G01^52^ were inserted into the murine IgG1 heavy chain (CH1 + Hinge + CH2 + CH3 regions) and the light chain (kappa), respectively, and both included the murine IgG heavy chain signal peptide (MGWSCIILFLVATATGVHS) on the N-terminus. The two genes were codon optimized for CHO cell expression and inserted into pcDNA3.4 using EcoR1 and Hind III sites following synthesis. Plasmids were then propagated in E. coli and used for the CHO cell transfection. The FNI-9 carrying the substitutions R27D or R27E was designed based on computational analysis (PDB: 8G3O) that indicated R27 in the light chain of FNI9 would likely be repelled by the E244K substitution in the NAs from recent H3N2 strains.

### Recombinant NA insect cell expression and purification

Recombinant NA (rNA) proteins were produced by infecting Sf9 insect cells with recombinant baculoviruses (BVs) that were generated using pFastBac vectors encoding the secreted rNA constructs. The His-tagged rN1/BR18 and rN2/KS17 were designed and purified as previously described^60,61^. The secreted rN1/Vic19, rN2/Dar21 and rNB/Austria21 constructs were all designed similar with a cleavable azurocidin signal peptide followed by a Strep-Tag, the tetrabrachion tetramerization domain (rN1/Vic19 and rN2/Dar21) or the vasodilator-stimulated phosphoprotein tetramerization domain (rNB/Austria21) and residues 35–469 of the NAs from A/Victoria/2570/2019 (H1N1) or A/Darwin/9/2021 (H3N2), or residues 39–466 from B/Austria/1359417/2021. Briefly, Sf9 cells were harvested 72 h post-infection, clarified by sequential sedimentations (10 min; 4000 × *g* and 30 min; 10,000 × *g*), concentrated 6-fold by tangential flow filtration (TFF) using a 30 kDa molecular weight cutoff (MWCO) Sartocon Slice 200 PES membrane (Sartorius), and diafiltrated using 5 volumes of binding buffer (30 mM HEPES pH 7.0, 150 mM NaCl, 1 mM CaCl_2_). Diafiltrated samples were purified by Strep-Tactin XT affinity chromatography (Cytiva) according to the manufacturer’s recommendations. Purified rNAs were diafiltrated by TFF using 30 kDa MWCO PES membrane with binding buffer, concentrations were determined by a *BCA assay* (Pierce), and rNAs were adjusted to ∼0.5–1.0 mg/ml prior to aliquoting and storage at −80 °C.

### NA monoclonal antibody hybridomas and antibody purification

All hybridomas were contracted out to Genscript. Briefly, mice were immunized three times with rN1/Vic19, rN2/Dar21, or rNB/Austria21 in the presence of adjuvant. Sera were tested for reactivity with the rNA antigen versus the two that were not used for immunization. Hybridomas from positive mice were generated and screened with the rNA antigen versus the two that were not used for immunization. Positive hybridomas were rescreened by ELLA and ELISA binding using viruses pre and post purification, respectively. Hybridomas that were positive in the rescreen for either ELISA alone or both ELISA and ELLA were selected, 2 clones of each were expanded, and the hybridoma media from the expansion were used in this study along with the antibodies purified from each selected clone with Protein A Sepharose.

### Chemical synthesis of sialoside-based NA inhibitor and streptavidin coupling

Synthesis of the biotinylated sialoside-based NA inhibitor 4-guanidino-Neu5Acα2–3 Galβ1–4GlcNAc–βProNH-PEG4-Biotin is described in another study^55^. The inhibitor was coupled to streptavidin (NAi + SA) by mixing in a 1:2 molar ratio (0.159 mM inhibitor and 16.8 mg/ml streptavidin) and incubating 1 h at 37 °C prior to use.

### NA titration using MUNANA

All MUNANA assays were performed in 96-well low protein binding black clear bottom plates in duplicate. A 4-methyl umbelliferone (MU) standard was diluted to 0.2 mM in reaction buffer (25 mM MES pH 7, 150 mM NaCl, 1 mM CaCl_2_, 1% BSA) with or without 0.025% TX100 as indicated for the samples and used to make seven additional two-fold serial dilutions in reaction buffer. The eight standards were transferred to a column (25 μl/well) and mixed with 37 °C reaction buffer (75 μl/well). Test viruses in allantoic fluid or recombinant NA were serially diluted 1:2 in 37 °C reaction buffer with or without 0.025% TX100 as indicated and added to the same plate (25 μl/well). Samples were mixed with 37 °C reaction buffer (50 μl/well) to a volume of 75 μl, reactions were initiated by adding 37 °C reaction buffer containing 0.4 mM MUNANA (25 μl/well) and the fluorescence (Ex λ: 355 nm, Em λ: 450 nm) was measured on a Cytation 5 (Biotek) plate reader at 37 °C for 10 min using 30 s intervals. Activities were determined by dividing the slope of the linear region of the emission (RFU) versus time graph by the slope of the RFU versus MU pmol/well standard, and the dilutions that cleaved ~0.1–0.4 pmol/s of MUNANA were used for all subsequent analysis. For endpoint analysis all wells received 100 μl of stop solution (0.133 mM Glycine, 0.06 M NaCl, 0.083 N Na_2_CO_3_, pH 10.7) after the indicated incubation time, and the fluorescence was measured.

### Generation and analysis of NA inhibition curves

Purified NAi-MAbs, synthesized NAi alone, and the NAi coupled to Streptavidin (NAi + SA) were serially diluted in reaction buffer with or without 0.025% TX100 and added (50 µl/well) to a 96-well plate in duplicate. Test viruses diluted to the target NA activity levels in reaction buffer with or without 0.025% TX100 were added (25 µl/well) to all wells mixed and incubated for 3 h unless specified otherwise at 37 °C. Reactions were initiated by adding (25 µl/well) of reaction buffer with or without 0.025% TX100 and 0.4 mM MUNANA, and the fluorescence was generally read by a kinetic and 10 min endpoint analysis. NA activity measurements were normalized by the NA activity in control wells without inhibitor which was set to 100%, and concentrations are with respect to the final 100 µl reaction. NA activity measurements were plotted with respect to the inhibitor concentration in GraphPad Prism 8.0, and a variable slope four-parameter least squares regression analysis was performed to determine the half-maximal inhibitory concentration (*IC*_*50*_) and the Hill slope (H). Concentrations corresponding to the *IC*_*80*_ were calculated based on the following formula where F corresponds to the desired inhibitory concentration: *IC*_*F*_ = *IC*_*50*_ × (F/(100-F))^(1/H)^.

### NA active site proximity assay (NASPA) and titer determination

Sera, hybridoma media, or purified MAbs were serially diluted in reaction buffer with or without 0.025% TX100 and added (25 µl/well) to a 96-well plate in duplicate. Control wells containing serial dilutions of hybridoma medium, negative sera or buffer, and the MU standard were included. Test viruses were diluted to the target NA activity levels in reaction buffer with or without 0.025% TX100 and added (25 µl/well) to all wells except the standard, and the plate was incubated for 1 h at 37 °C. The NAi-MAb, NAi, or NAi + SA were diluted to an ~IC_80_ in reaction buffer with or without 0.025% TX100 and added (25 µl/well) to all wells except the standard. The plate was incubated for 3 h at 37 °C after which reactions were initiated by adding (25 µl/well) of 0.4 mM MUNANA in reaction buffer with or without 0.025% TX100 that was prewarmed to 37 °C and the fluorescence was read directly by a kinetic or by endpoint analysis after incubation for 10 min or 2 h at 37 °C and the addition of stop solution (100 µl/well). The mean activity and standard deviation (SD) of the control wells were determined, and titers corresponded to the largest dilution factor where both samples showed an activity level above the mean plus three SDs of the control wells (steric inhibition) or below the mean minus three SDs (active site inhibition).

### NA titer determination by MUNANA

The set up for the assay was identical to NASPA except the NAi-MAb was omitted. Briefly, sera or hybridoma medium was serially diluted in reaction buffer with 0.025% TX100 and added to the plate (25 µl/well). Control wells were included that contained serial dilutions of hybridoma medium or buffer and the MU standard. Test viruses diluted in reaction buffer with 0.025% TX100 were added (25 µl/well) and incubated for 1 h at 37 °C. Reactions were initiated by adding (50 µl/well) of 0.2 mM MUNANA in reaction buffer containing 0.025% TX100 that was prewarmed to 37 °C, and the fluorescence was read by a kinetic or endpoint analysis after incubation for 10 min or 2 h at 37 °C and the addition of stop solution. The mean activity and standard deviation (SD) of the control wells were determined, and titers corresponded to the largest dilution factor where both samples showed activity levels below the control mean minus three SDs.

### Enzyme linked immunosorbent assay (ELISA) titer determination

Immunol 2HB 96-well plates were coated (100 µl/well) overnight at 4 °C with purified egg-propagated B/Austria21, Vic19 (H1N1) or Dar21 (H3N2) viruses diluted (10 µg/ml) in 1X coating buffer. Plates were washed 3 × 200 µl/well with PBS pH 7.4 and blocked for 1 h at 37 °C with PBS pH 7.4 containing 1% BSA (200 µl/well). Plates were washed 3 × 200 µl/well with PBS pH 7.4, clarified hybridoma medium serially diluted in PBST with 0.1% BSA was added to each well (100 µl/well), and the plate was incubated 1 h at 37 °C. Plates were washed 3 × 200 µl/well with PBST, and an HRP-conjugated goat-anti-mouse IgG secondary antibody diluted (1:20,000) in PBST containing 0.1% BSA was added (100 µl/well). Plates were incubated 1 h at 37 °C, washed 4 × 200 µl/well with PBST, and developed with OPD for 10 min at 37 °C. Reactions were stopped by adding 50 µl/well of 2 N sulfuric acid, and the Abs at 490 nm was read using a Cytation 5 (Biotek) plate reader.

### Human sera analysis

All human sera were from a previous healthy volunteer challenge study that was performed at the NIH Clinical Center after participants signed an informed consent form^28^. Human sera were heat inactivated by incubating at 55 °C for 30 min prior to being serially diluted in reaction buffer containing 0.025% TX100. The dilution series was then used in the NASPA and MUNANA activity assay, both with a 2 h substrate (MUNANA) incubation time at 37 °C and an endpoint analysis following the addition of stop solution. For protein A/G depletions, 10 ml of a 20% slurry consisting of 10% Protein A Agarose beads and 10% Protein G Agarose beads or Agarose beads alone were washed 3 × 10 ml with reaction buffer containing 0.025% TX100 and each one was finally resuspended in 4 ml reaction buffer containing 0.025% TX100 to create a 50% slurry. Serum diluted 1:20 in reaction buffer (200 µl per sample) was added to tubes containing 200 µl of the Protein A/G Agarose bead slurry or the control Agarose bead slurry and rotated overnight at 37 °C. Beads were sedimented (4000 × g; 5 min), and the supernatant was serially diluted in reaction buffer containing 0.025% TX100. Serial dilutions were then used in the NASPA and MUNANA activity assay as described above. Assays were performed with clarified H6N1/CA09 virus grown in SPF eggs and the H1N1/CA09 challenge virus grown in MDCK cells. ELLA titers for the human sera presented in this manuscript were from an analysis performed in a previous study^28^.

### Statistical analysis

Statistical analysis was performed with Excel (mean and standard deviation) and GraphPad Prism 10 software.

## Supplementary information

Supplementary Information

## Data Availability

All data supporting the findings of this study are available within the Article and the Supporting Information files. Any unique materials used in the study are available from the corresponding author on request.
