# Supplementary material for: Influenza neuraminidase active site proximity assay for rapid profiling of inhibitory antibodies and antigenic drift
Source: NPJ Vaccines. 2025 Jun 7;10:118. doi: 10.1038/s41541-025-01173-2 (PMC12145422; doi:10.1038/s41541-025-01173-2)
Supplement: Supplementary file 1 — Supplementary Information [file 41541_2025_1173_MOESM1_ESM.pdf]

Supplementary Table 1 | HI sera and MAb titers against H1N1/CA09 and H6N1/CA09 viruses

Antisera/MAb	H1N1/CA09	H6N1/CA09
H6N1/CA09 Ferret antisera	<20	80
H6N1/CA09 Mouse#1 antisera	<20	160
H6N1/CA09 Mouse#2 antisera	<20	80
H1 MAb (B11)*	640	<20
H1 MAb (6G)*	2560	<20
H1 MAb (C19)*	640	<20

*Initial monoclonal antibody (MAb) concentration was 1 mg/ml

Supplementary Table 2 | Genetic compositions of the H6 reassortant viruses

Strain Name	NA strain soure	HA strain soure	Internal genes
**H6N1/BR07**	A/Brisbane/59/2007 (H1N1)	A/turkey/MA/3740/1965 (H6N2)	PR8
**H6N1/CA09**	A/California/04/2009 (H1N1)	A/turkey/MA/3740/1965 (H6N2)	PR8
**H6N1/MI15**	A/Michigan/45/2015 (H1N1)	A/turkey/MA/3740/1965 (H6N2)	PR8
**H6N1/BR18**	A/Brisbane/02/2018 (H1N1)	A/turkey/MA/3740/1965 (H6N2)	PR8
**H6N1/Vic19**	A/Victoria/2570/2019 (H1N1)	A/turkey/MA/3740/1965 (H6N2)	PR8
**H6N2/HK14**	A/HongKong/4801/14 (H1N2)	A/turkey/MA/3740/1965 (H6N2)	PR8
**H6N2/Dar21**	A/Darwin/9/2021 PR8 (H3N2)	A/turkey/MA/3740/1965 (H6N2)	PR8
**H6N1/MI15**	A/Michigan/45/2015 (H1N1)	A/turkey/MA/3740/1965 (H6N2)	PR8
**H6NB/Austria21**	B/Austria/1359417/2021*	A/turkey/MA/3740/1965 (H6N2)	PR8
**H6NB/Phu13**	B/Phuket/3073/2013*	A/turkey/MA/3740/1965 (H6N2)	PR8

*These NAs were chimeras consisting of residues 34-466 from the indicated influenza B strains fused to the NA residues 1-35 from the H1N1 strain A/Brisbane/59/2007.

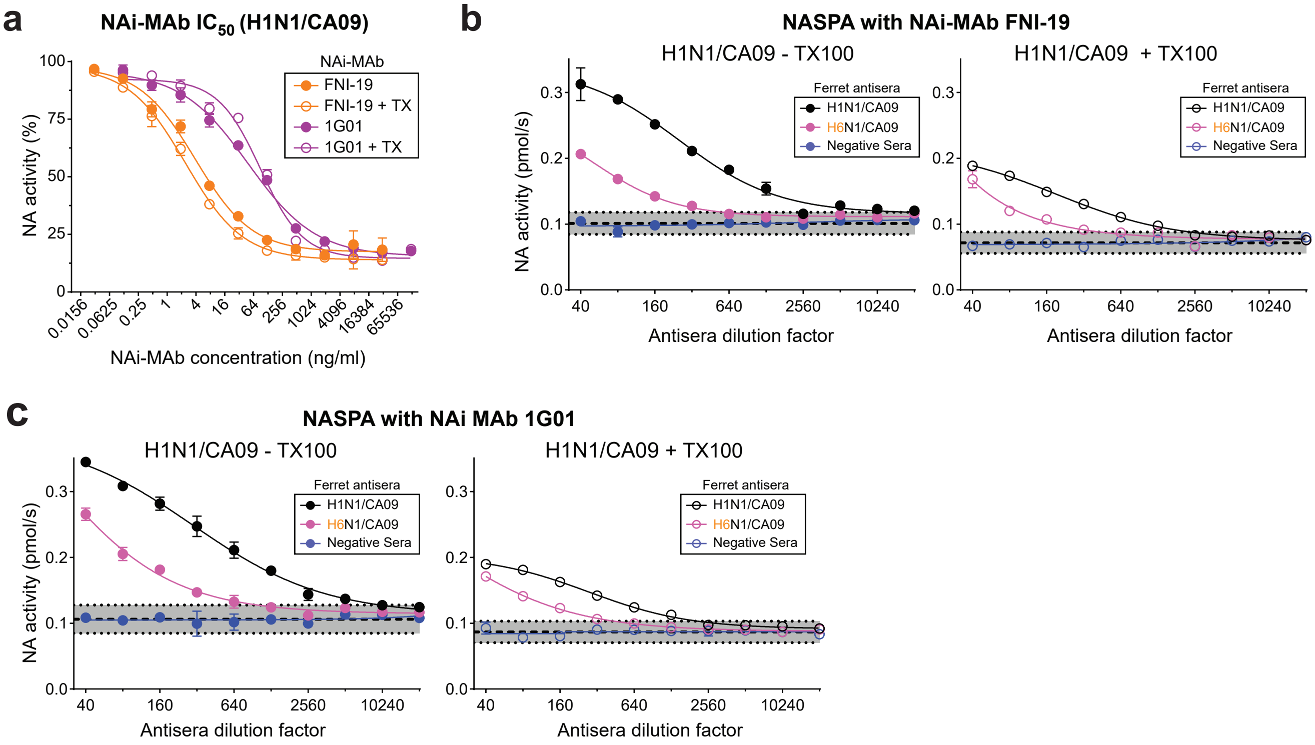

Supplementary Figure 1 | NASPA results with different monoclonal antibodies that bind the NA active site. a. Inhibition curves for the NAi-MAbs FNI-19 and 1G01 with untreated and TX100-treated H1N1/CA09 virus are shown. The indicated NAi-MAb amounts were incubated with the virus for 3 h prior to measuring NA activity by MUNANA. **b and c.** NA activity data is displayed from NASPA that was performed using the NAi-MAbs (**b**) FNI-19 and (**c**) 1G01 with untreated and TX100-treated H1N1/CA09 virus and the indicated ferret antisera. Assays were performed in duplicate using NAi-MAb amounts approximating the IC_{80} and are displayed as the mean ± SD. NA activities were read for 10 min after MUNANA addition. Grey regions indicate the mean (dashed line) ± 3 SDs (dotted line) that were determined using negative control ferret sera.

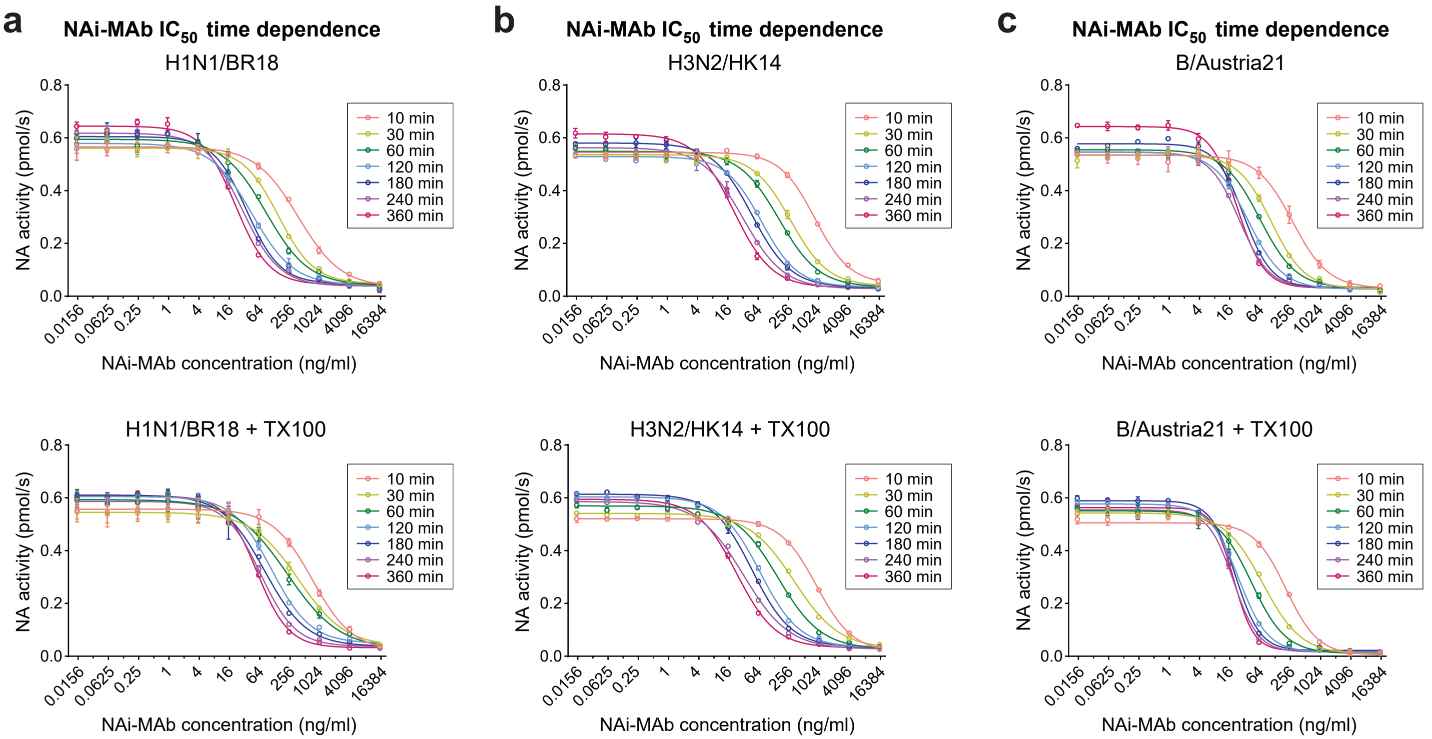

Supplementary Figure 2 | Time-dependent variation in the NAi-MAb (FNI-9) binding to NAs. a-c. NAi-MAb (FNI-9) inhibition curves obtained after the indicated incubation times at 37 °C with the vaccine viruses (**a**) H1N1/BR18, (**b**) H3N2/HK14, and (**c**) B/Austria21 are displayed. Measurements were performed using virus (upper panels) and TX100-treated virus (lower panels). Assay were run in duplicate and displayed as the mean ± SD. NA activities were measured for 10 min after MUNANA addition.

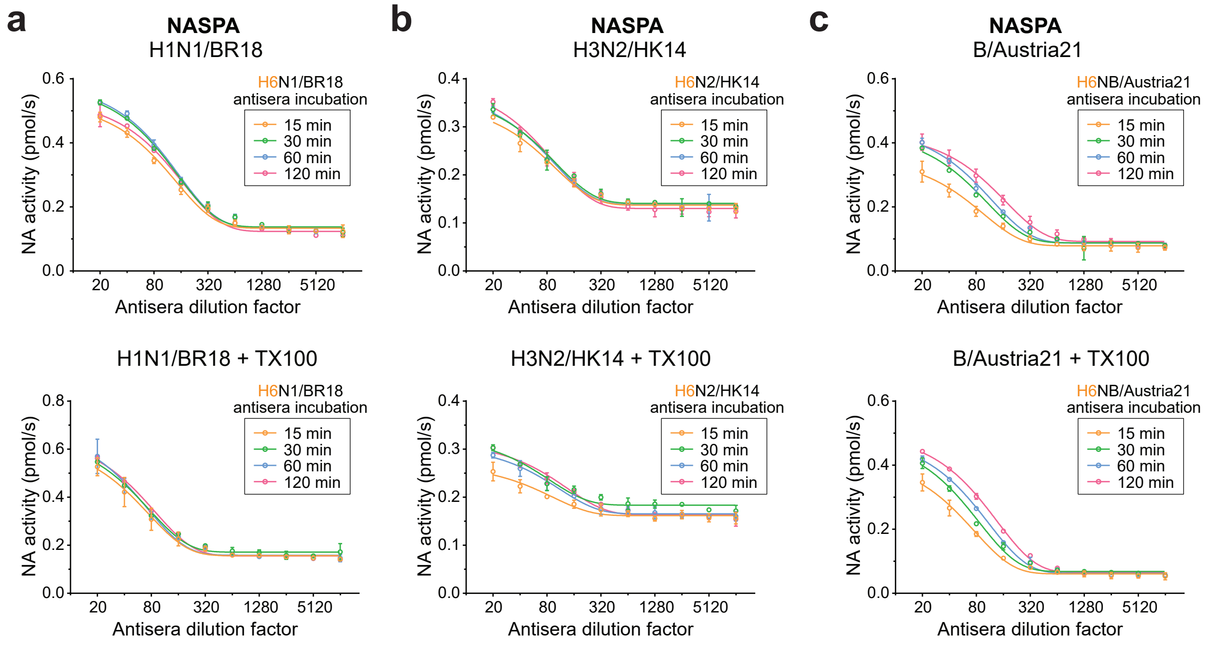

Supplementary Figure 3 | NASPA results from vaccine viruses with different ferret antisera incubation times. a-c. NASPA results of the untreated and TX100 treated vaccine viruses (**a**) H1N1/BR18, (**b**) H3N2/HK14, and (**c**) B/Austria21 incubated with ferret antisera for the indicated times at 37 °C are shown. Assays were performed in duplicate using NAi-MAb amounts approximating the IC_{80} and are displayed as the mean ± SD. NA activities were measured for 10 min after MUNANA addition.

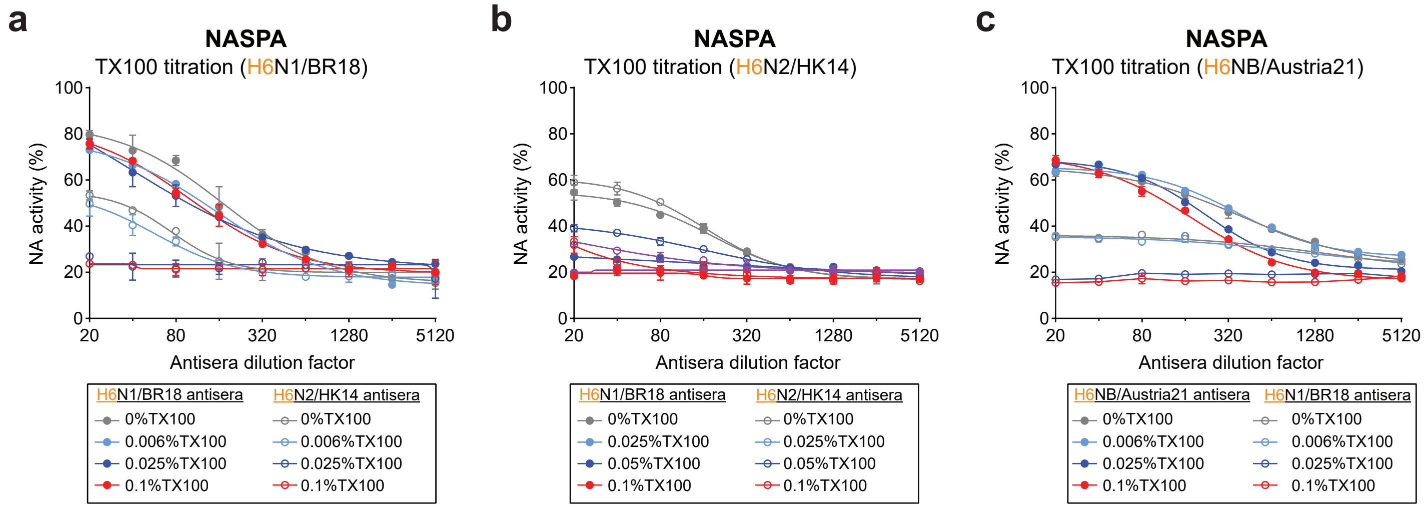

Supplementary Figure 4 | Influence of TX100 on NASPA results from ferret antisera that recognize HA in the test virus. a-c. NASPA was performed with H6 reassortant viruses carrying NAs from the vaccine strains (**a**) H1N1/BR18, (**b**) H3N2/HK14, and (**c**) B/Austria21 in the presence of increasing TX100 concentrations (values correspond to final concentrations in the 100 µl reaction) and the indicated ferret antisera. Assays were performed in duplicate using NAi-MAb amounts approximating the IC_{80} and are displayed as the mean ± SD. NA activities were measured for 10 min after MUNANA addition. Percent NA activities were calculated using control wells that did not receive the NAi-MAb.

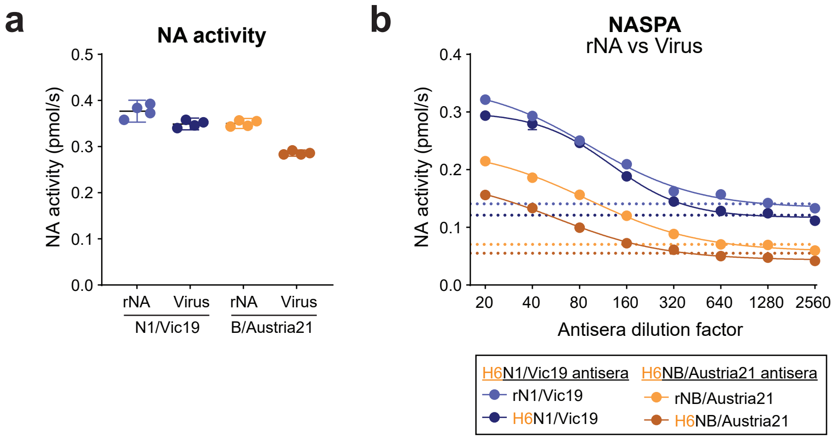

Supplementary Figure 5 | NASPA results comparing viral and recombinant NAs. a. NA activities of the indicated recombinant NA (rNA) or TX100-treated virus samples that were used for NASPA. rNAs and the viruses corresponded to the H1N1 vaccine strain Vic19 (N1/Vic19) or the type B vaccine strain B/Austria21. **b.** NASPA results from the indicated rNAs and TX100-treated H6 reassortant viruses incubated with ferret antisera against either H6N1/Vic19 or H6NB/Austria21 are shown. Assays were performed in duplicate using NAi-MAb amounts approximating the IC_{80} and are displayed as the mean ± SD. NA activities were measured for 10 min after MUNANA addition. Dotted lines correspond to the mean+3SD of the mock control wells for each sample.

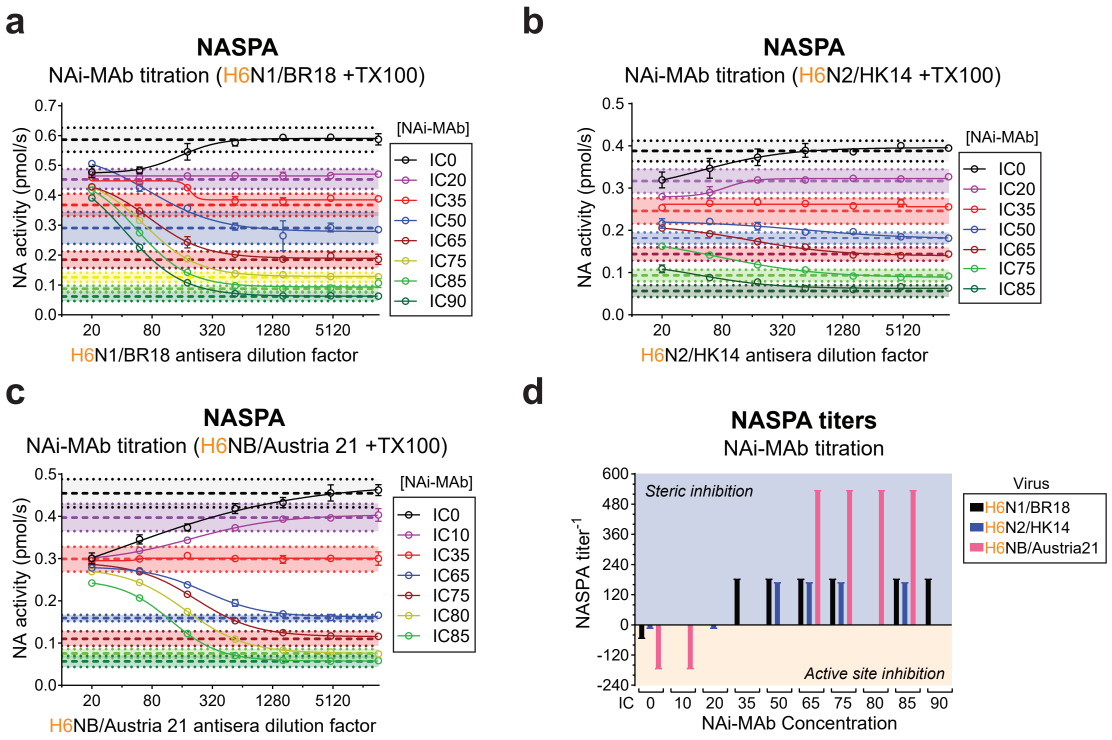

Supplementary Figure 6 | Identification of optimal NAi-MAb concentration for NASPA. a-c. NASPA results are displayed for TX100 treated H6 reassortant viruses carrying NAs from the vaccine strains (**a**) H1N1/BR18, (**b**) H3N2/HK14, and (**c**) B/Austria21 that were incubated with the indicated NAi-MAb (FNI-9) concentrations for 3 h at 37 °C prior to the addition of ferret antisera. Assays were performed in duplicate and are displayed as the mean ± SD. NA activities were measured for 10 min after MUNANA addition. Highlighted regions for each NAi-MAb concentration corresponds to the mean (dashed line) ± 3 SDs (dotted line) of mock control wells. **d.** NASPA NAI titers obtained for each virus with the indicated inhibitory concentration (IC) of the NAi-MAb (FNI-9) are shown. NASPA titers correspond to the largest dilution factor that was above (positive titers) or below (negative titers) 3SDs of the control wells.

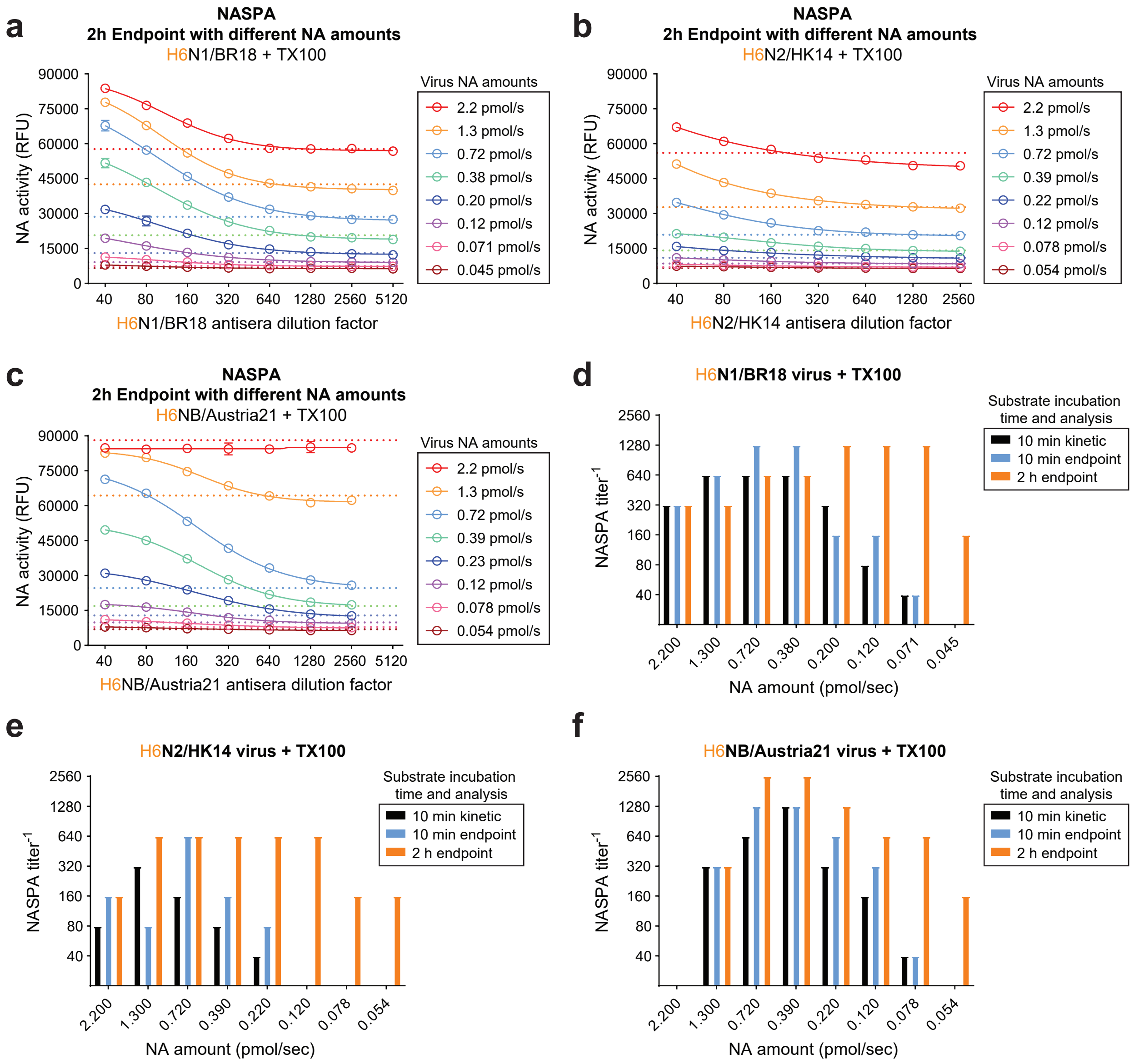

Supplementary Figure 7 | Influence of NA amounts and substrate incubation times on NASPA results. a-c. Results from NASPA performed with TX100-treated (**a**) H6N1/BR18, (**b**) H6N2/HK14, and (**c**) H6NB/Austria21 reassortant virus samples with the indicated NA activities and ferret antisera are displayed. Data were collected after a 2 h incubation with MUNANA at 37 °C. Dotted lines correspond to the mean+3SD of the mock control wells for the different NA amounts. **d-f.** NASPA titers determined using the TX100-treated (**d**) H6N1/BR18, (**e**) H6N2/HK14, and (**f**) H6NB/Austria21 reassortant virus samples with the indicated NA activities and matching ferret antisera are displayed. Titers were collected for each sample by measuring NA activities for 10 min after MUNANA addition (kinetic), after a 10 min incubation with MUNANA (endpoint) or after a 2 h incubation with MUNANA (endpoint).

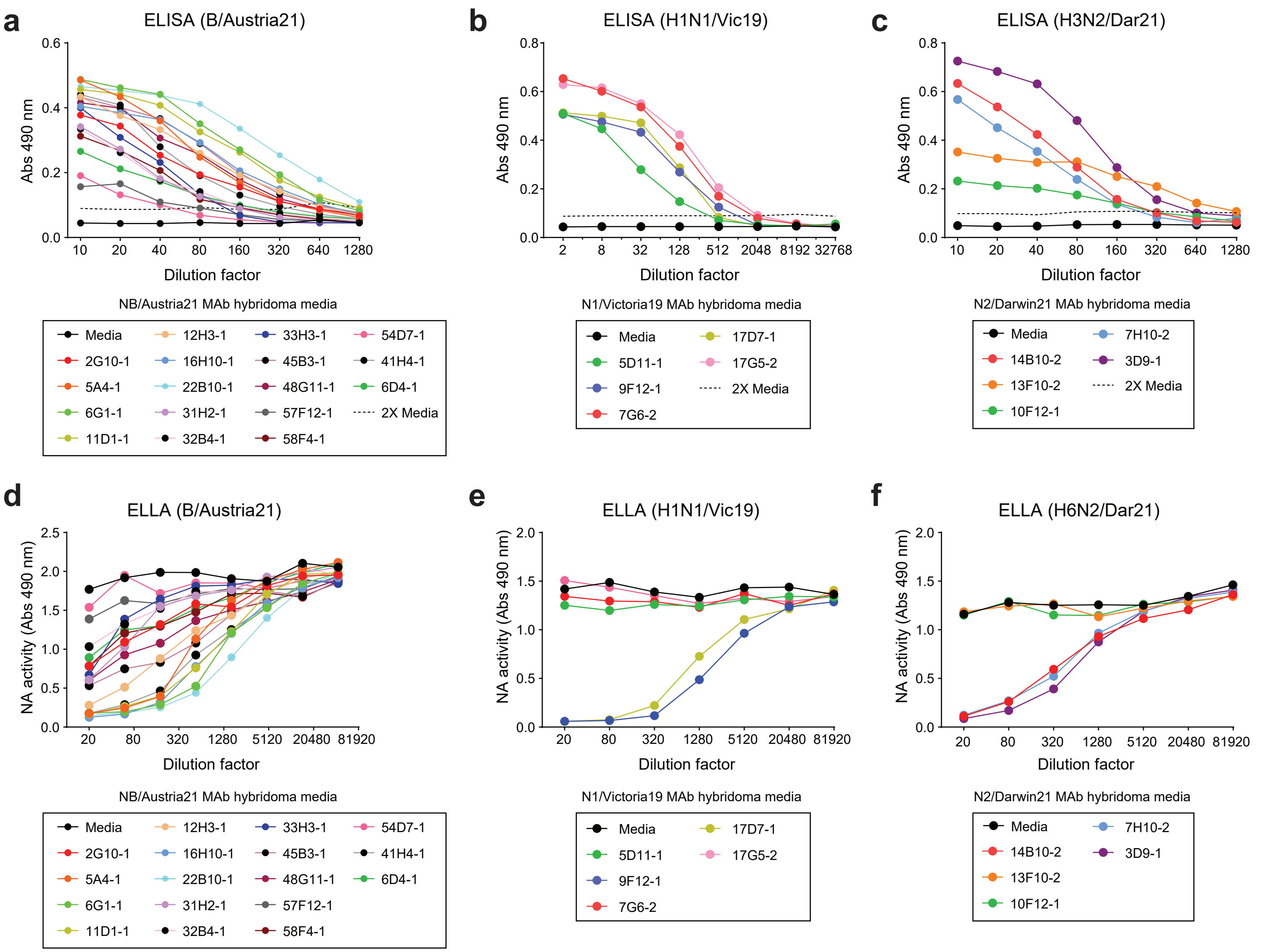

Supplementary Figure 8 | ELISA and ELLA results for the NA MAb panel. a-c. ELISA results from the indicated NA MAb hybridoma media are shown. Wells were coated with either 1 µg of purified (**a**) B/Austria21 virus, (**b**) H1N1 virus (Vic19) or (**c**) H3N2 virus (Dar21). ELISA endpoint titers corresponded to the largest dilution factor that was 2-fold higher than the medium only control (black dashed lined). **d-f.** ELLA results for the indicated NA MAb hybridoma media are shown. ELLA was performed using allantoic fluid containing either (**d**) B/Austria21 virus, (**e**) H1N1 virus (Vic19) or (**f**) H3N2 virus (Dar21). ELLA titers corresponded to the dilution factor that resulted in ~50% loss of NA activity.

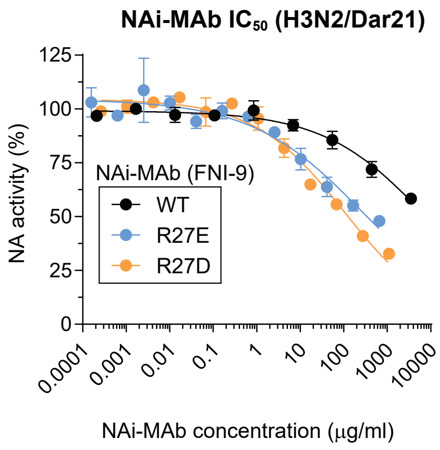

Supplementary Figure 9 | Inhibition curves of the NAi-MAb FNI-9 and two modified variants with H3N2 Dar21. Dar21 virus treated with TX100 was incubated with the indicated amounts of the NAi-MAb FNI-9 or the two modified variants (R27D and R27E) for 3 h at 37 °C prior to measuring the NA activity with MUNANA for 10 min.

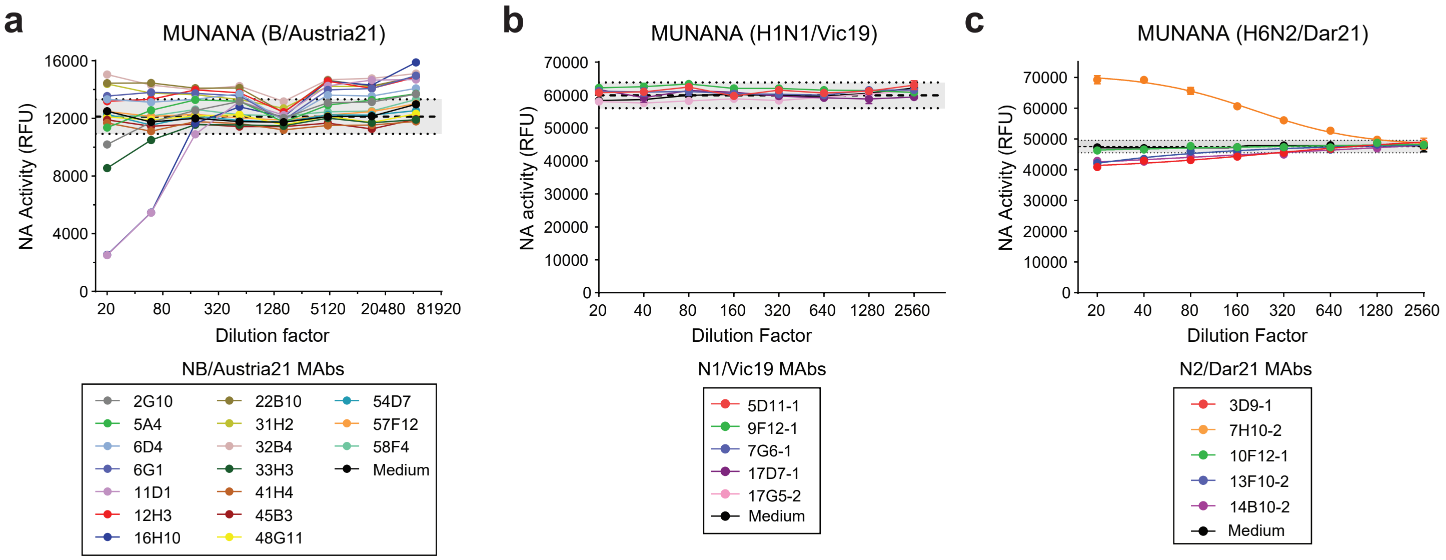

Supplementary Figure 10 | Influence of the NA MAbs on NA active site activity. a-c. NA MAb hybridoma media were serially diluted and incubated with either TX100-treated (**a**) B/Austria21 virus, (**b**) H1N1 virus (Vic19) or (**c**) H6N2Dar21 virus for 1 h at 37 °C prior to incubating with MUNANA and taking an endpoint reading. Grey region indicates the mean (dashed line) ± 3 SDs (dotted line) of the mock control wells with medium.

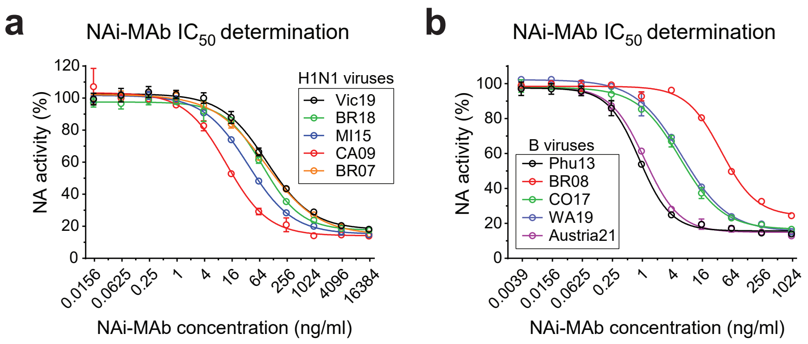

Supplementary Figure 11 | NAi-MAb IC_{50} determinations for NAs from the H1N1 and type B vaccine strains. NAi-MAb (FNI-9) inhibition curves with the indicated (**a**) H1N1 and (**b**) type B vaccine strains are displayed. The assays were performed with TX100-treated incubated with the indicated NAi-MAb concentrations for 3 h at 37 ℃ prior to measuring NA activity with MUNANA.

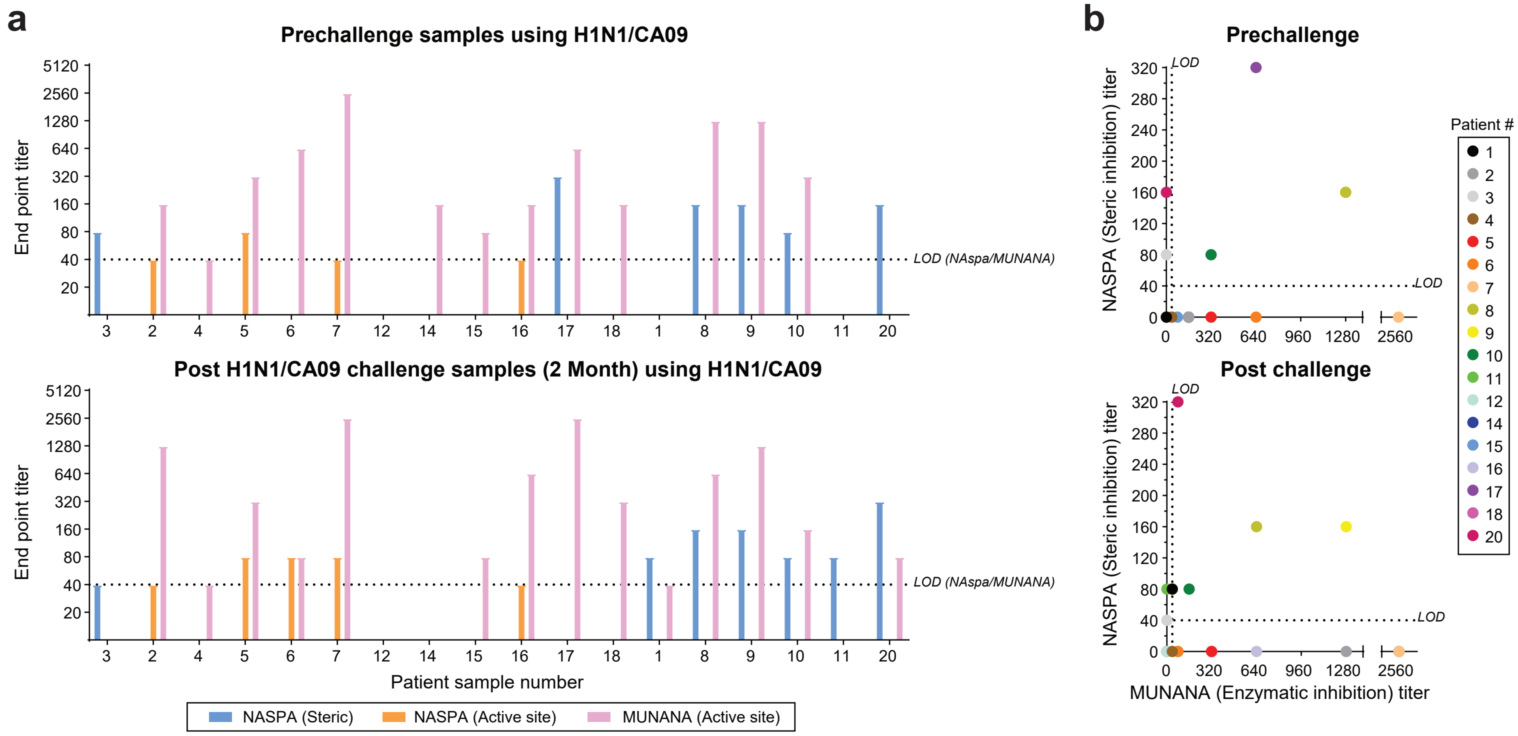

Supplementary Figure 12 | NASPA analysis of human NA responses pre- and post-challenge with H1N1/CA09. a. Endpoint titers determined with NASPA and a MUNANA activity assay for the indicated patients pre-challenge (upper panel) and 2 months post-challenge (lower panel) are displayed. Data was obtained using TX100-treated H1N1/CA09 challenge virus grown in MDCK cells **b.** Pre- (upper panel) and post-challenge (lower panel) correlation plots showing the steric inhibition and NA active site inhibition endpoint titers obtained from each serum by NASPA and the MUNANA activity assay, respectively. Limits of detections (LODs) for the two assays are shown by dotted lines. Values below the LOD were assigned 0.

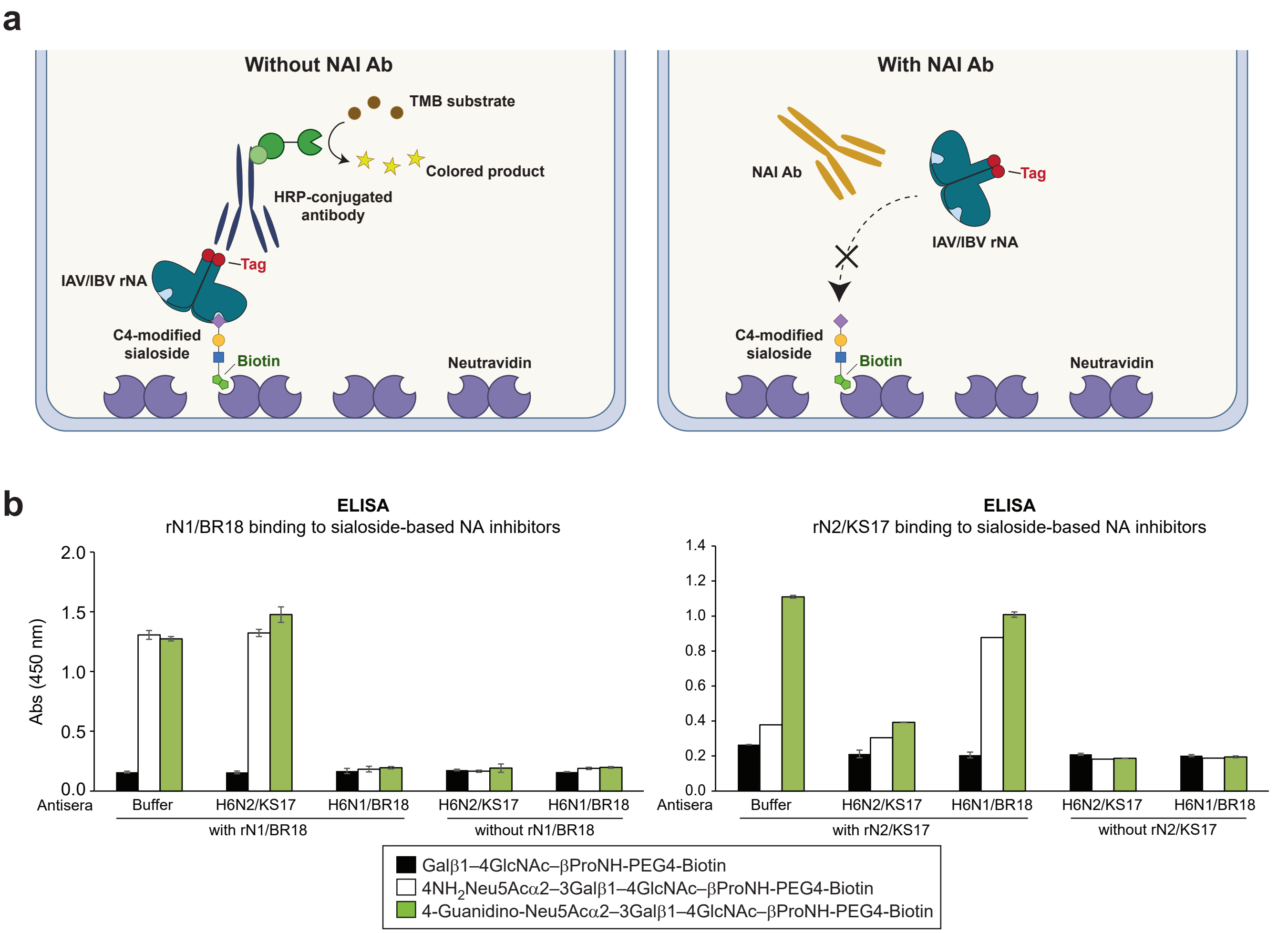

Supplementary Figure 13 | Ferret antisera inhibits NA binding to a sialoside-based NA inhibitor in an ELISA strategy. a. Illustration of an ELISA strategy to measure NA binding to biotinylated sialosides in the absence and presence of NAI antibodies in ferret antisera, respectively. **b.** Indicated biotinylated sialosides (10 μM, 20 μl) containing a terminal α2–3-linked 4-guanidino-Neu5Ac or 4NH2Neu5Ac and a negative control glycoside Galβ1–4GlcNAc–βProNH-PEG4-Biotin were incubated in 384-well neutravidin-coated plates at 4 °C overnight. Plates were washed and blocked with 1% ovalbumin in PBS buffer containing 0.05% tween-20 prior to adding recombinant (left panel) N1/BR18 or (right panel) N2/KS17 (0.5 μg/ml, 20 μl) that was preincubated with ferret antisera raised against H6N1/BR18 or H6N2/KS17 viruses (10-fold dilution) for 30 min at 37 °C. Plates were incubated at 4 °C for 24 h, washed and NA binding was detected by incubating with a horse radish peroxidase (HRP)-conjugated anti-His6 mouse monoclonal antibody for 30 min at 4 °C. Wells were washed and developed for 30 min at room temperature with 3,3’,5,5’-tetramethylbenzidine (TMB) and reactions were stopped with 2 M sulfuric acid prior to reading the absorbance at 450 nm. Samples containing no ferret antisera or no recombinant NA were used as controls.

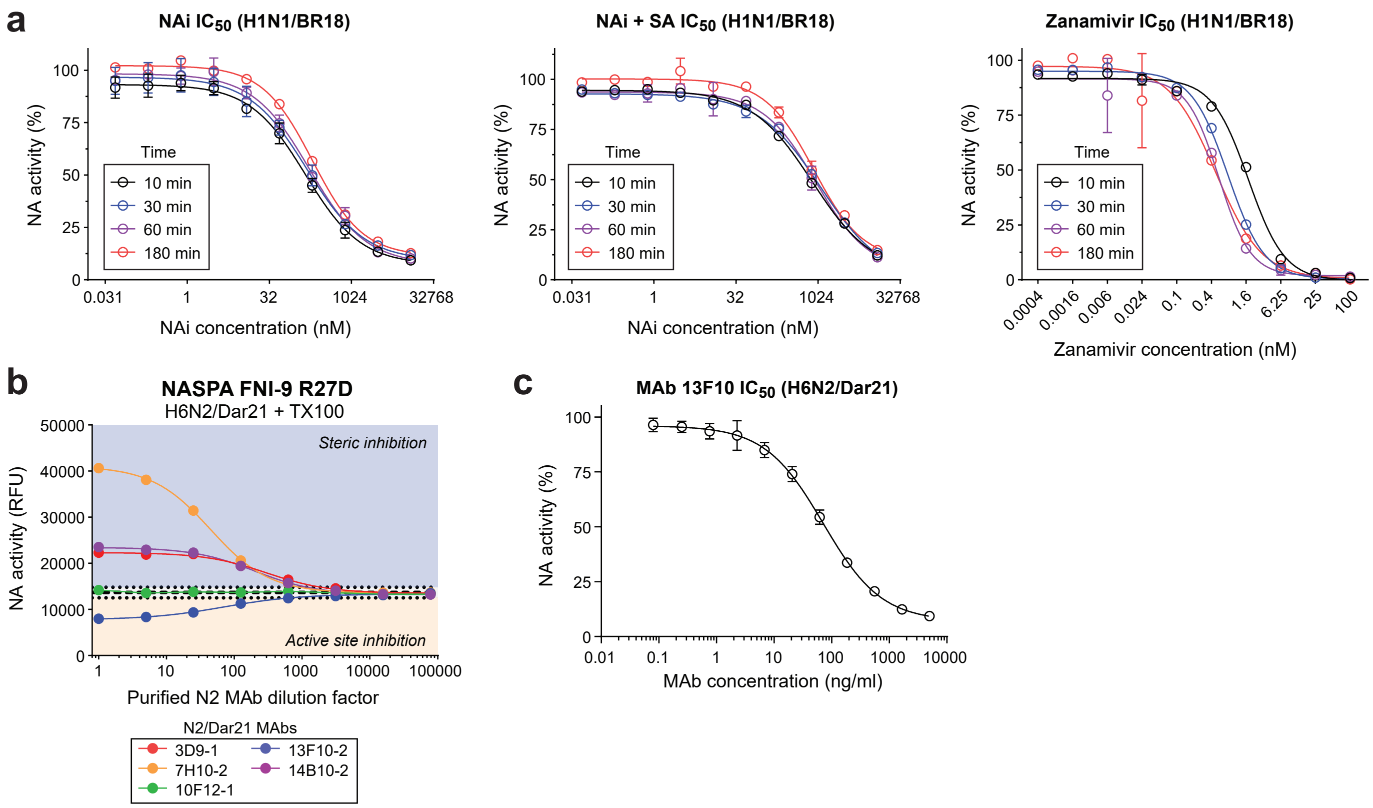

Supplementary Figure 14 | Inhibition curves for the synthetic NAi, synthetic NAi ± SA, Zanamivir, and MAb 13F10 with the indicated viruses. a. Inhibition curves for the NAi (left panel), NAi + SA mixed at a 1:2 molar ratio (middle panel), and Zanamivir (right panel) after the indicated incubation times with the H1N1/BR18 virus at 37 °C are displayed. **b.** NASPA data obtained with TX100-treated H6N2/Dar21 virus and the indicated amounts of the purified N2 MAbs. Data was collected in duplicate and a single representative curve for each MAb is shown. The modified NAi-MAb (FNI-9 R27D) was used at an ~IC_{80}. Endpoint NA activities (RFU) were measured after a 10 min incubation with MUNANA at 37 °C. Grey regions indicate the mean ± 3 SDs (dotted line) from the control reactions containing PBS. **c.** TX100-treated H6N2/Dar21 virus was incubated with the indicated N2 MAb 13F10 concentrations for 1 h at 37 °C prior to measuring NA activity with MUNANA.
